# An immunotherapy guide constructed by cGAS-STING signature for breast cancer and the biofunction validation of the pivotal gene HOXC13 via *in vitro* experiments

**DOI:** 10.3389/fimmu.2025.1586877

**Published:** 2025-08-08

**Authors:** Zhenling Dai, Ziqun Gu, Rongrong Shen, Jingshuai Wang

**Affiliations:** ^1^ Department of General Surgery, Shanghai Sixth People’s Hospital, Shanghai, China; ^2^ Department of Breast Surgery, International Peace Maternal and Child Health Hospital of China Welfare Society, Shanghai, China; ^3^ Department of Obstetrics and Gynecology, Shanghai East Hospital, Tongji University School of Medicine, Shanghai, China

**Keywords:** HOXC13, immunotherapy, cGAS-STING, subgrouping, breast cancer, Support Vector Machine (SVM), RandomForest (RF), eXtreme Gradient Boosting (XGB)

## Abstract

**Objective:**

As the most common cancer in women, immunotherapy has become a pivotal element in the treatment of breast cancer, particularly for cases resistant to traditional therapies. The cyclic GMP-AMP synthase (cGAS)-stimulator of interferon genes (STING) pathway is recognized as the primary DNA-sensing mechanism that initiates immune and inflammatory responses. In this study, we aim to explore the role of the cGAS-STING pathway in breast cancer immunotherapy resistance.

**Methods:**

Multiple machine learning algorithms were applied to construct an immunotherapy subgroup model and *in vitro* experiments were performed to verify the HOXC13 in regulating BRCA immunity.

**Results:**

Building upon extensively researched genes within the cGAS-STING pathway, we identified eight genes that serve as indicators of breast cancer’s responsiveness to anti-PD1 therapy. Through consensus clustering, patients were categorized into high-response and low-response groups based on these eight genes. Subsequently, we extracted the pivotal gene set by WGCNA, which showed the highest correlation with the response to immune therapy, followed by the selection of 11 genes, which held significant associations with T-cell exhaustion, immune score, and patient survival. Employing machine learning, our novel classification model based on the 11-gene signature effectively differentiated between high-response and low-response groups in 16 out of 18 independent breast cancer cohorts from the GEO database. Notably, this 11-gene signature also predicted the sensitivity of breast cancer to both conventional and immune therapies, aligning closely with predictions from the OncoPredict algorithm. Further, *in-vitro* experiments confirmed the regulatory role of HOXC13, one of the 11 genes, in the cGAS-STING pathway. Moreover, miR-26a-5p, a microRNA previously identified as a suppressor in breast cancer, was demonstrated to regulate HOXC13.

**Conclusion:**

Our study implies that HOXC13 is a potential therapy target for BRCA immunotherapy and 11-gene signature is a potential tool for clinical evaluation of anti-PD1/PDL1 therapy efficacy.

## 1 Introduction

Breast cancer (BC), the most prevalent cancer among women, can be classified into various subtypes: luminal A, luminal B, HER2-enriched, and triple-negative breast cancer (TNBC). These classifications are based on the expression of estrogen receptor (ER), human epidermal growth factor receptor 2 (HER2), progesterone receptor (PR), and Ki-67 (1, 2). Patients with BC may experience significant benefits from traditional surgical treatment, radiotherapy, targeted therapy, and chemotherapy, while others may require immunotherapy, which yields varying responses. For example, patients with HER2 amplification have seen substantial improvements in survival rates due to HER2-targeted therapies such as trastuzumab (3, 4). Conversely, patients with luminal A (ER-positive and/or PR-positive, Ki-67-positive) or B subtypes (ER-positive and/or PR-positive, Ki-67-negative) often benefit from endocrine therapies like tamoxifen (5). For those with TNBC or those who develop resistance to initial treatments, combining immunotherapy may be the final viable option (6). Since immunotherapy has become a pivotal element in the treatment of breast cancer, particularly for cases resistant to traditional therapies, identifying the specific group of breast cancer patients who can benefit from immunotherapy is crucial.

The cyclic GMP-AMP synthase (cGAS)-stimulator of interferon genes (STING) pathway is recognized as the primary DNA-sensing mechanism that initiates inflammatory and immune responses. Recent studies suggest that the cGAS-STING pathway can be both a potent inducer of immune responses and a contributor to immune suppression, depending on the context (7–10). In breast cancer, the activation of the cGAS-STING pathway plays a complicated role. Some researchers suggested that its activation could suppress breast cancer progression, while some studies showed that this pathway was beneficial to its survival and metastasis (11–14). It suppresses tumors by activating type I interferons (IFNs), triggered by cytosolic DNA from chromosomal instability (CIN) or therapy-induced damage. This enhances dendritic cell infiltration and CD8^+^ T cell activation, boosting antitumor immunity. Tumor-derived DNA or cGAMP also stimulates IFN release in immune cells like NK cells (15–21). Conversely, in high-CIN tumors, such as triple-negative breast cancer, persistent cGAS-STING signaling promotes progression via NF-κB-mediated IL-6 production, STAT3 activation, and PD-L1 upregulation, aiding immune evasion (14, 22, 23). Further research is needed to clarify this pathway’s complex dynamics.

In this study, we explored the role of the cGAS-STING pathway in breast cancer immunotherapy resistance, particularly focusing on the pivotal role of the gene HOXC13. Using bioinformatics tools, we analyzed gene expression profiles to construct an immune-response classification model, while validating these findings through *in vitro* experiments. Additionally, we investigated the regulatory influence of microRNAs, such as miR-26a-5p, on the HOXC13 gene, shedding light on its potential as a therapeutic target.

## 2 Methods

All data utilized in this study were carefully curated and verified for accuracy. The TCGA and GEO datasets were independently processed and cross-validated to ensure consistency. All experiments and bioinformatics analyses were independently performed by at least two researchers to confirm reproducibility and reliability. Data discrepancies were resolved through collaborative discussions, and the final results represented a consensus between independent analyses.

### 2.1 Data sources

The Cancer Genome Atlas (TCGA) Breast Carcinoma (BRCA) dataset was accessed via the UCSC Xena platform (https://xenabrowser.net/). This dataset included RNA sequencing-based gene expression profiles and matched clinical information from 1,078 breast cancer patients. Data preprocessing involved log-transformation and normalization using the edgeR package in R. For external validation, 18 datasets from the Gene Expression Omnibus (GEO) database were used. These included GSE21653 (267 samples for validation of gene expression and drug sensitivity), GSE42568 (104 samples for survival data and prognostic evaluation), GSE145668 (110 samples for immune checkpoint expression and analysis of PD1/PD-L1), GSE162228 (126 samples for survival outcomes and therapy response), and GSE20685 (328 samples for immune profiling and clinical outcomes). Additional datasets included GSE45255 (130 samples for immune infiltration), GSE88770 (118 samples for survival data), GSE97342 (91 samples for disease-free survival), GSE12093 (137 samples for recurrence data), GSE11121 (85 samples for response rates), GSE20711 (140 samples for prognosis), GSE20681 (141 samples for biomarker validation), GSE42568 (104 samples for survival analysis), GSE162228 (126 samples for clinical response), GSE31348 (82 samples for immune checkpoint expression), GSE31448 (95 samples for immune markers), and GSE45255 (108 samples for immune response). Each dataset was carefully selected based on relevance to drug sensitivity, immune infiltration, therapy response, or survival outcomes, ensuring comprehensive coverage of breast cancer features.

### 2.2 Bioinformatics analysis

**Drug sensitivity prediction:** Drug sensitivity was predicted using the OncoPredict package in R, which modeled tumor drug response based on genomic and transcriptomic features. TCGA-BRCA served as the training cohort, while datasets such as GSE21653 and GSE20685 were used for validation. Drugs analyzed included epirubicin (EPI), cyclophosphamide (CTX), paclitaxel (PTX), docetaxel (DTX), cisplatin (CDDP), gemcitabine (GEM), olaparib (OLA), and tamoxifen (TAM). Statistical comparisons of drug responses between high-response (H-Response) and low-response (L-Response) groups were performed using Wilcoxon rank-sum tests.

**Anti-PD1 therapy response prediction:** Predictions for anti-PD1 therapy response were made using the Tumor Immune Dysfunction and Exclusion (TIDE) algorithm (http://tide.dfci.harvard.edu). Immune cell infiltration was quantified using the CIBERSORT and xCell algorithms. TIDE scores, along with exclusion and dysfunction scores, were used to classify response groups. Immune checkpoint pathways, including PD1/PD-L1 and T-cell receptor signaling, were evaluated through pathway enrichment analysis conducted using the GSVA and clusterProfiler packages.

**Molecular subtype identification:** Non-negative matrix factorization (NMF) and consensus clustering were used to identify molecular subtypes within TCGA-BRCA. These subtypes were validated across GEO datasets using Kaplan-Meier survival plots and immune feature characterizations.

Differential Gene Expression and Pathway Enrichment: Differentially expressed genes were identified using DESeq2, and pathway enrichment was performed with clusterProfiler and WikiPathway, highlighting immune-related signaling and cancer progression pathways.

**Machine learning:** Support Vector Machine(SVM), RandomForest(RF), eXtreme Gradient Boosting(XGB), Generalized Linear Model(GLM), Gradient Boosting Machine(gbm), K-Nearest Neighbors(KKNN), Neural Network(NNET), Least Absolute Shrinkage and Selection Operator(LASSO) were applied in this study, and each algorithm used 10-fold repeated cross-validation. Here, algorithms from R including rf, svmRadial, xgbDART, glm, gbm, kknn, glmnet were applied, and all of them were performed by “*caret”* package. In machine learning part, TCGA cohort was applied as training cohort, and 70% of which was intra-training subgroup, and other 30% was intra-testing subgroup. Details about the R codes were in **Supplementary Files**.

**Meta-analysis:** Hazard ratios were calculated through meta-analyses performed using the meta package in R, pooling data from TCGA and GEO cohorts.

### 2.3 Experimental methods

**Cell culture:** Breast cancer cell lines MDA-MB-231 and BT549 were obtained from ATCC and maintained in DMEM or RPMI-1640 supplemented with 10% fetal bovine serum (FBS), 1% penicillin-streptomycin, insulin, and incubated at 37°C in a humidified atmosphere with 5% CO2. All cell lines were controlled the number of passages within 20–30 times.

**Peripheral blood mononuclear cell extraction:** Peripheral blood mononuclear cells (PBMCs) were isolated from healthy donors. After diluting blood with sterile PBS, it was carefully layered onto a density gradient medium (Ficoll-Paque) and centrifuged at 400–500g for 30 minutes. The mononuclear cell layer was then aspirated, and washed with PBS to remove contaminants, and the cell count and viability were assessed. Short-term storage involved suspending PBMCs in a medium with 10–20% FBS at 4°C. For long-term preservation, cells were cryopreserved in 90% FBS and 10% DMSO. Aliquots were frozen at a controlled rate to −80°C and then transferred to liquid nitrogen. PBMCs were not permitted to amplified.

**HOXC13 Knockdown and miR-26a-5p Mimic Transfection:** BRCA cell lines (BT549 and MBA-MD-231) were transplanted in 6-cells plate for 24h (5 × 10^5^ cells). Following, refreshed the culture with OPTI-MEM (FBA-free) for 2 hours. For HOXC13 knockdown experiments, siRNA targeting HOXC13 was transfected into cells using Lipofectamine 3000 following the manufacturer’s instructions. Transfection complexes were prepared by diluting siRNA (50 nM) and Lipofectamine 3000 in Opti-MEM medium, incubated at room temperature for 15 minutes, then added dropwise to cells. After 48 hours of incubation, cells were harvested for downstream assays. For overexpression studies, miR-26a-5p mimics were transfected similarly, using scrambled RNA as a control. The HOXC13 knockdown efficiency was verified by western blot, and the results were showed in **Figure 10A**.

**Figure 1:**
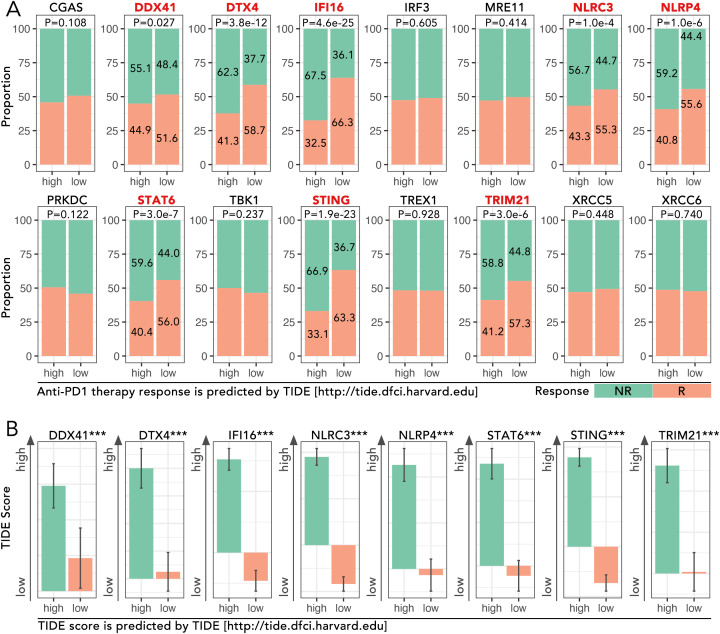
Analysis of cGAS-STING pathway genes in TCGA-BRCA cohort and their association with immunotherapy response predicted by TIDE. **(A)** Proportions of predicted response (R) and non-response (NR) to anti-PD1 therapy based on high and low expression levels of cGAS-STING pathway genes, which were obtained from the GSEA Molecular Signatures Gene Set Database (MSigDB) v7.1. The expression level of DDX41, DTX4, IFI16, NLRC3, NLRP4, STAT6, STING and TRIM21 potentially determined the anti-PD1/PDL1 efficacy (Chi-square Test, p<0.05). **(B)** TIDE (Tumor Immune Dysfunction and Exclusion) scores for the above 8 genes were analyzed, and the high-expression group of each mentioned gene was accompanied by a high TIDE score (Mann-Whitney U test, ***p < 0.001). The TIDE analysis and response predictions were conducted using the TIDE framework [http://tide.dfci.harvard.edu].

**Western blotting:** Cells were lysed in RIPA buffer containing protease and phosphatase inhibitors. Lysates were clarified by centrifugation at 12,000 × g for 15 minutes at 4°C, and protein concentrations were measured using a BCA assay. Equal protein amounts (10 µg) were separated by SDS-PAGE and transferred to PVDF membranes. Membranes were blocked in 5% non-fat milk in TBS-T for 1 hour at room temperature, followed by overnight incubation with primary antibodies at 4°C. Primary antibodies were from Proteintech and Cell Signaling Technology, including HOXC13 (Proteintech, 11408-1-AP), JAK3 (Proteintech, 66287-1-Ig), phosphorylated JAK3 (Proteintech, 29101-1-AP), STAT1 (CST, 14994), and phosphorylated STAT3 (Tyr705, CST, 9145), among others. Following washing, membranes were incubated with HRP-conjugated secondary antibodies for 1 hour. Bands were visualized using enhanced chemiluminescence and quantified with ImageJ.

**Flow cytometry:** Peripheral blood mononuclear cells (PBMCs) were isolated from healthy donor blood using Ficoll-Paque density gradient centrifugation. PBMCs were co-cultured with HOXC13 knockdown or control breast cancer cells at a 10:1 ratio in RPMI-1640 with 10% FBS. After 48 hours, cells were harvested, stained with fluorophore-conjugated antibodies from BioLegend, including CD8 (BioLegend, 344714) and PD1 (BioLegend, 329906), and analyzed on a BD FACSCanto II flow cytometer. Data were analyzed with FlowJo software.

**Immunofluorescence staining:** Formalin-fixed paraffin-embedded (FFPE) tissue sections were deparaffinized, rehydrated, and subjected to antigen retrieval in citrate buffer (pH 6.0) at 95°C for 20 minutes. After blocking with 5% BSA, sections were incubated overnight at 4°C with primary antibodies against HOXC13 and CD8. The next day, sections were washed and incubated with Alexa Fluor-conjugated secondary antibodies for 1 hour at room temperature. Nuclei were counterstained with DAPI, and images were captured using a Leica confocal microscope. The colocalization of markers was analyzed using ImageJ.

**Transwell migration and EdU proliferation assays:** For migration assays, BRCA cell lines were treated with different, followed by PBS washing, and then digested with EDTA-containing trypsin for 2 minutes, neutralizing the trypsin with complete medium. Centrifuge at 100g for 5 minutes, collect the cell pellet, resuspend the cells in medium containing 2% FBS, and adjust the cell concentration to 4 × 10^5^ cells/ml. Trans-well cells were pre-treated with PBS for 2 hours. After that, 100ul 4 × 10^5^ cells/ml cells were seeded in the upper chamber of transwell inserts with an 8-µm pore size. The lower chamber contained a medium supplemented with 10% FBS as a chemoattractant. After 24 and 48 hours, non-migrated cells on the upper surface were removed and migrated cells on the lower surface were fixed with 4% paraformaldehyde, stained with 0.1% crystal violet, and counted under a microscope. For proliferation assays, cells with different treatments were washed with PBS 5 mins for twice, followed by being incubated with 10 µM EdU for another 2 hours, fixed with 4% paraformaldehyde, and stained using a Click-iT EdU imaging kit. Fluorescence signals were detected with a confocal microscope, and images were analyzed using ImageJ.

### 2.4 Statistical analysis

All statistical analyses were conducted in R (v4.2.0). Kaplan-Meier survival analyses were performed using the survival and survminer packages, with log-rank tests assessing differences between groups. ROC curves and AUC values were computed using the pROC package to evaluate model performance. Meta-analyses of hazard ratios were performed using the meta package, applying random-effects models to account for heterogeneity across studies. The statistical method for comparing data between two groups used an independent samples t-test or a non-parametric test (Mann-Whitney U), while the statistical method for comparing data among three or more groups used a non-parametric test (Wilcoxon). Tabular data comparisons between two or more groups used the Chi-Square test (Fisher’s Exact Test). Statistical significance was defined as P < 0.05, with significance levels indicated in figures as *P < 0.05, **P < 0.01, and ***P < 0.001.

## 3 Results

### 3.1 cGAS-STING pathway genes and immunotherapy response

The expression levels of cGAS-STING pathway genes, including DDX41, DTX4, IFI16, NLRC3, NLRP4, STAT6, STING, and TRIM21, were analyzed in the TCGA-BRCA cohort to evaluate their roles in predicting response to anti-PD1 therapy. High-expression groups for these genes consistently exhibited higher proportions of non-responders (NR) compared to low-expression groups. For instance, in DTX4, the proportion of NR was 62.3% in the high-expression group compared to 41.3% in the low-expression group, showing a significant difference (p = 3.8e-12) (**Figure 1A**). Similarly, for STING, NR accounted for 58.7% in the high-expression group versus 36.6% in the low-expression group (p < 0.001) (**Figure 1A**). TIDE scores, which predict immune dysfunction, were significantly higher in high-expression groups. For example, the mean TIDE score for DTX4 was 0.85 in the high-expression group versus 0.45 in the low-expression group (p < 0.001) (**Figure 1B**). These findings implied the potential of cGAS-STING pathway genes in driving immunotherapy resistance.

### 3.2 Enhanced molecular subtyping via consensus clustering in TCGA-BRCA compared to NMF

**Figure 2:**
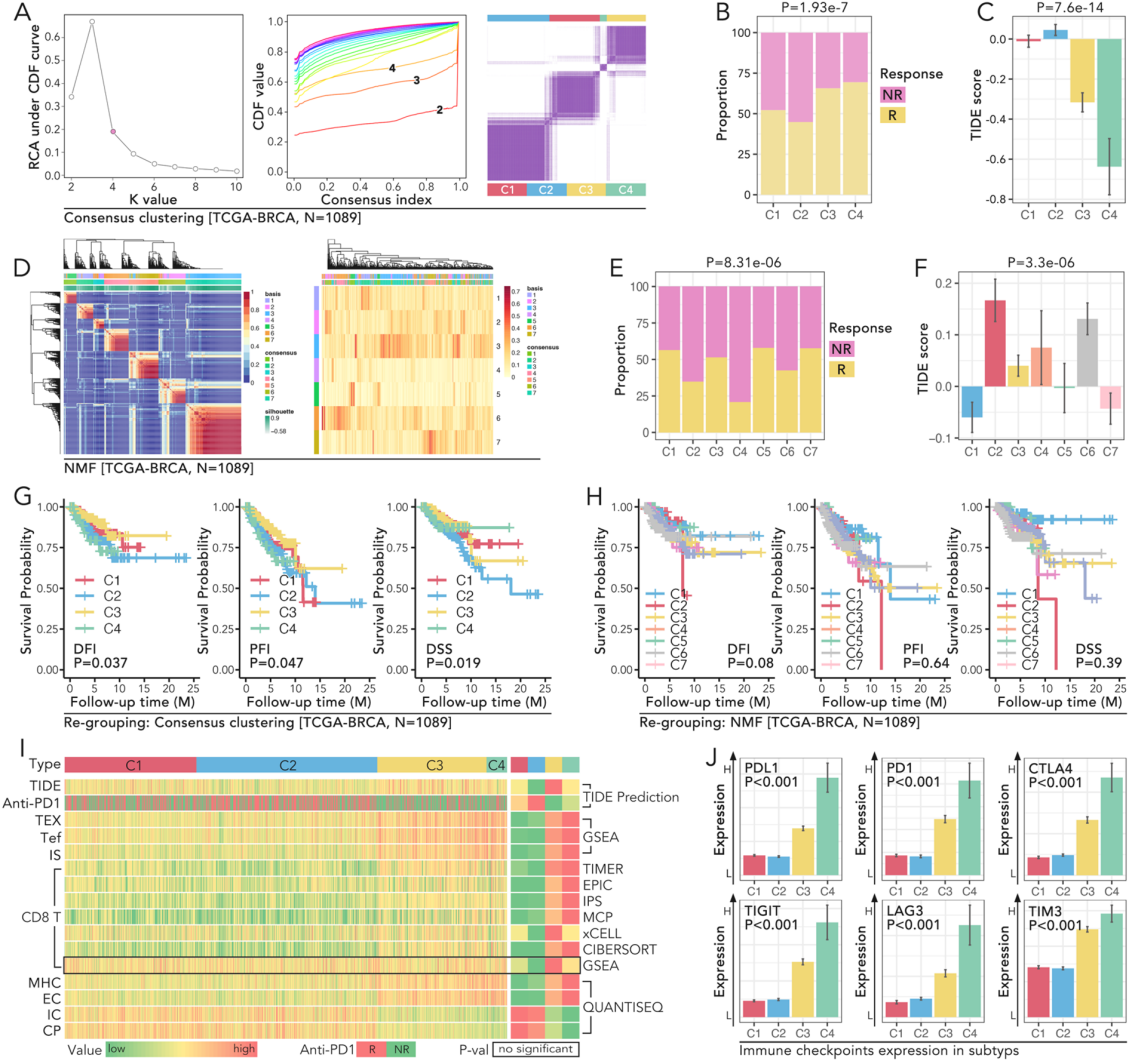
Molecular subtyping of TCGA-BRCA reveals distinct immune profiles, prognostic significance, and immune checkpoint expression patterns. **(A)** Consensus clustering of TCGA-BRCA cohort (N=1089) to identify molecular subtypes. The relative change in area under the CDF curve was used to determine the optimal number of clusters. CDF curves for k=2 to 10 were displayed, and the consensus matrix for k=4 is the best selection. **(B, C)** Comparison of immune response and TIDE scores amongst the four identified clusters (C1-C4) in the TCGA-BRCA cohort, in which **(B)** shows the proportion of non-responders (NR) and responders (R) in each cluster (P=1.93e-7), while **(C)** shows the TIDE score differences (P=7.6e-14). **(D–F)** NMF clustering of TCGA-BRCA cohort (N=1089), in which **(D)** displays the heatmap of clustering results with samples grouped into seven subtypes (C1-C7), and **(E)** shows the immune response proportions among these subtypes (P=8.31e-06), and **(F)** illustrates TIDE score variations (P=3.3e-06). **(G, H)** Kaplan-Meier survival curves of PFI (progression-free interval), DFI (disease-free interval), and DSS (disease-specific survival) for the subtypes (C1-C4), in which **(G)** all three prognoses hold significant statistic results (log rank p<0.05) in consensus clustering identified subtypes, while **(H)** no significance was observed in NMF identified subtypes (log rank P>0.05). **(I)** Heatmap representation of immune-related features across the four subtypes. All parameters but GSEA predicted CD8 T cell infiltration proportion held non-significance amongst subtypes(C1-C4). **(J)** Expression of immune checkpoints (PD1, PDL1, CTLA4, TIGIT, LAG3, TIM3) across the four consensus subtypes (C1-C4), and subtype C4 held the highest expression level of those six genes (Mann-Whitney U test, p<0.05).

Consensus clustering of gene expression data from the TCGA-BRCA cohort (n=1089) identified four distinct molecular subtypes: C1-C4 (**Figure 2A**). Among these, subtype C4 exhibited the most immune-excluded phenotype, with 76.1% of patients classified as non-responders (NR) to anti-PD1 therapy, compared to other subtypes (p = 1.93e-7) (**Figure 2B**). TIDE scores, which assess immune exclusion and dysfunction, were significantly higher in subtype C4 which was consensus with (p = 7.6e-14) (**Figure 2C**). These findings highlighted subtype C4 might not respond to the anti-PD1 therapy relatively. In addition to consensus clustering, seven subtypes (C1-C7) were identified by non-negative matrix factorization (NMF) (**Figure 2D**). Among these, subtype C7 exhibited the highest proportion of responders (R) to anti-PD1 therapy (72.4%) and the lowest TIDE scores, indicating minimal immune dysfunction. Subtype C4, on the other hand, remained the most immune-excluded (p = 8.31e-6 for response proportions; p = 3.3e-6 for TIDE scores) (**Figures 2E, F**). Survival analysis based on consensus clustering revealed significant differences in prognosis across the subtypes. Subtype C4 had the poorest progression-free interval (PFI), with a median of 28 months, compared to 65 months in C1 (p < 0.05) (**Figure 2G**). Similarly, Kaplan-Meier analyses for disease-free interval (DFI) and disease-specific survival (DSS) confirmed these prognostic differences among consensus clustering-defined subtypes. In contrast, survival analysis of subtypes defined by NMF clustering did not yield statistically significant differences (**Figure 2H**). To further characterize the immune landscape across the consensus clustering-defined subtypes, immune-related features were analyzed. A heatmap representation demonstrated significant differences in most immune-related parameters across subtypes (**Figure 2I**). Analysis of immune checkpoint expression levels revealed that subtype C4 exhibited the highest expression levels of key immune checkpoint molecules, including PD1, PDL1, CTLA4, TIGIT, LAG3, and TIM3 (**Figure 2J**). For example, PDL1 expression in C4 was 2.3-fold higher than in C1 (p < 0.05), and TIM3 was 1.9-fold elevated in C4 compared to C2 (p < 0.05) (**Figure 2J**). These elevated checkpoint levels further underscored the immune-suppressive microenvironment associated with subtype C4 and its potential as a target for checkpoint blockade therapies.

### 3.3 Identification of 11 tumor driver genes in BRCA via WGCNA

**Figure 3:**
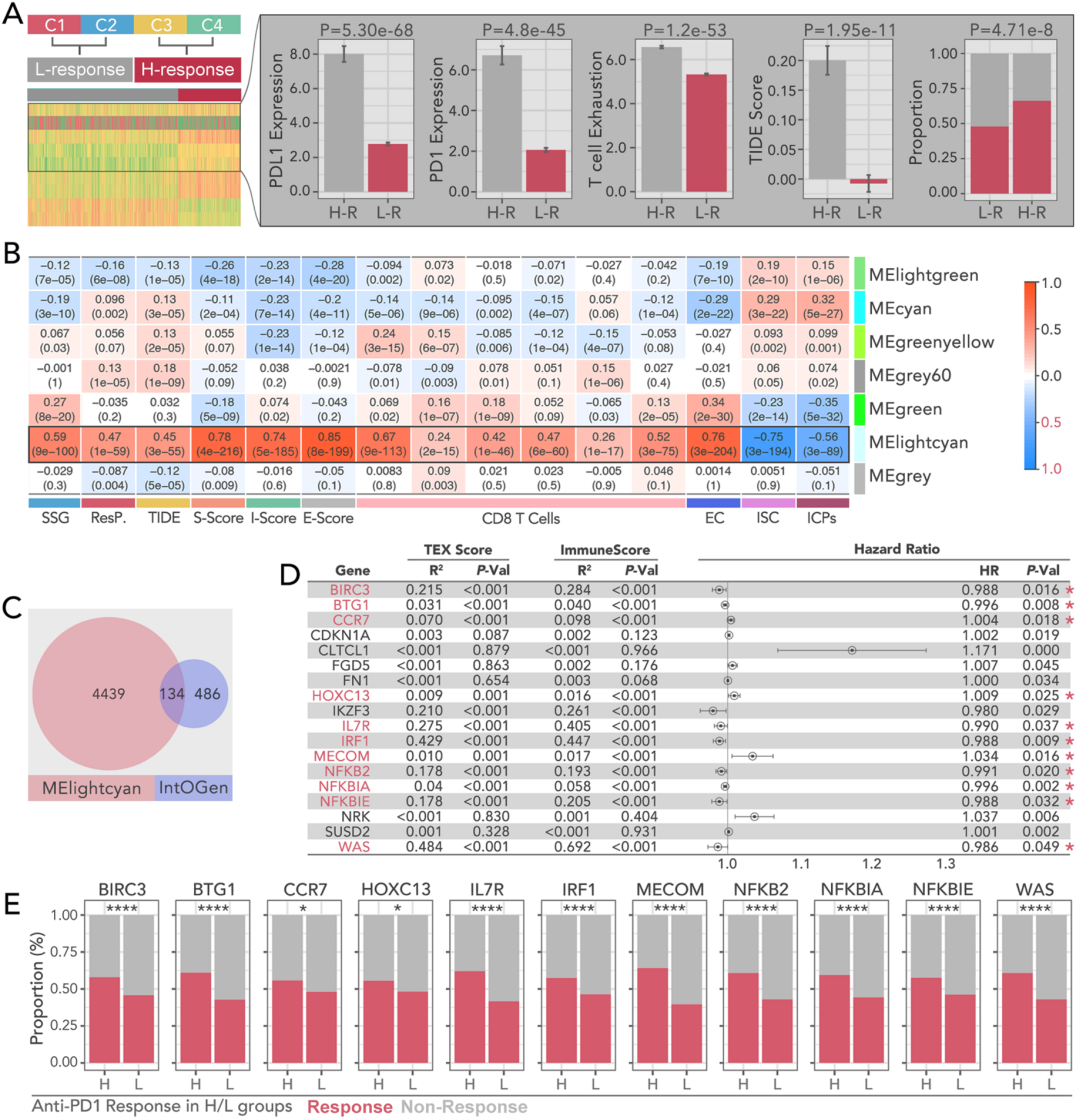
Prognostic and immunotherapy characteristics of WGCNA-derived bi-grouped subtypes in breast cancer. **(A)** Consensus clustering-based immunotherapy subtype identification, by which C1 and C2 were recognized as immunotherapy-sensitive subtypes (H-R), while C3 and C4 were recognized as non-sensitive subtypes (L-R). The higher level of PD-L, PD1, T cell exhaustion, and TIDE scores, and the lower level of immunotherapy response proportions, were the signatures in the H-R groups.(Chi-square Test was applied). **(B)** Correlation heatmap between module eigengenes (ME) and immune-related metrics, including S-score, TIDE, I-score, E-score, CD8 T cell infiltration, and immune-related features (EC, ISC, ICPs). And MElightcyan module is the optimal selection to collect the gene set, which holds the highest correlation. **(C)** Overlap of genes between the MElightcyan module and the IntOGen database and 134 shared genes were identified. **(D)** Hazard ratio analysis of pivotal genes identified from TEX (T cell exhaustion) and immune scores. Which, BIRC3, BTG1, CCR7, HOXC13, IL7R, IRF1, MECOM, NFKB2, NFKBIA, NFKBIE, and WAS were selected as candidates for further analysis. Genes significantly associated with prognosis are highlighted with red asterisks. (univariate cox regression test was applied to recognize prognosis-related genes). **(E)** Comparison of anti-PD1.PDL1 therapy response proportions in H-R and L-R groups for selected key genes (BIRC3, BTG1, CCR7, HOXC13, IL7R, IRF1, MECOM, NFKB2, NFKBIA, NFKBIE, WAS). (Chi-square Test was applied). * p<0.05, ****p<0.0001.

Based on the predicted anti-PD1 response, subgroups C1 and C2 identified by consensus clustering were defined as the low response (L-R) group, while C3 and C4 were defined as the high response (H-R) group (**Figure 3A**). Higher levels of PD-L1, PD1, T cell exhaustion, TIDE score, and antiPD1/PDL1 response rate were observed in the H-R subgroup (**Figure 3A**). Weighted Gene Co-expression Network Analysis (WGCNA) was performed to identify gene modules most closely related to immune response and subgroup classification. The MElightcyan module emerged as the most correlated with stromal score (r = 0.78, p < 0.001), immune score (r = 0.74, p < 0.001), and CD8+ T cell infiltration (r = 0.69, p < 0.001) (**Figure 3B**). Upon intersecting the 4,573 genes from the MElightcyan module with the 620 well-recognized tumor driver genes (24) (**Figure 3C**), we identified 134 overlapping genes. Among these, 11 genes—including BIRC3, BTG1, CCR7, HOXC13, IL7R, IRF1, MECOM, NFKB2, NFKBIA, NFKBIE, and WAS —emerged as key players in tumor progression and immune regulation. Hazard ratio (HR) analysis by univariate Cox regression revealed that a higher expression of BIRC3 (HR = 0.994, p = 0.002), BTG1 (HR = 0.99, p = 0.005), and CCR7 (HR = 1.01, p < 0.001) was associated with significantly worse prognosis (**Figure 3D**). Anti-PD1 response analysis showed a marked difference between high- and low-expression groups (**Figure 3E**).

### 3.4 Spatial distribution of the 11 genes in the tumor microenvironment

**Figure 4:**
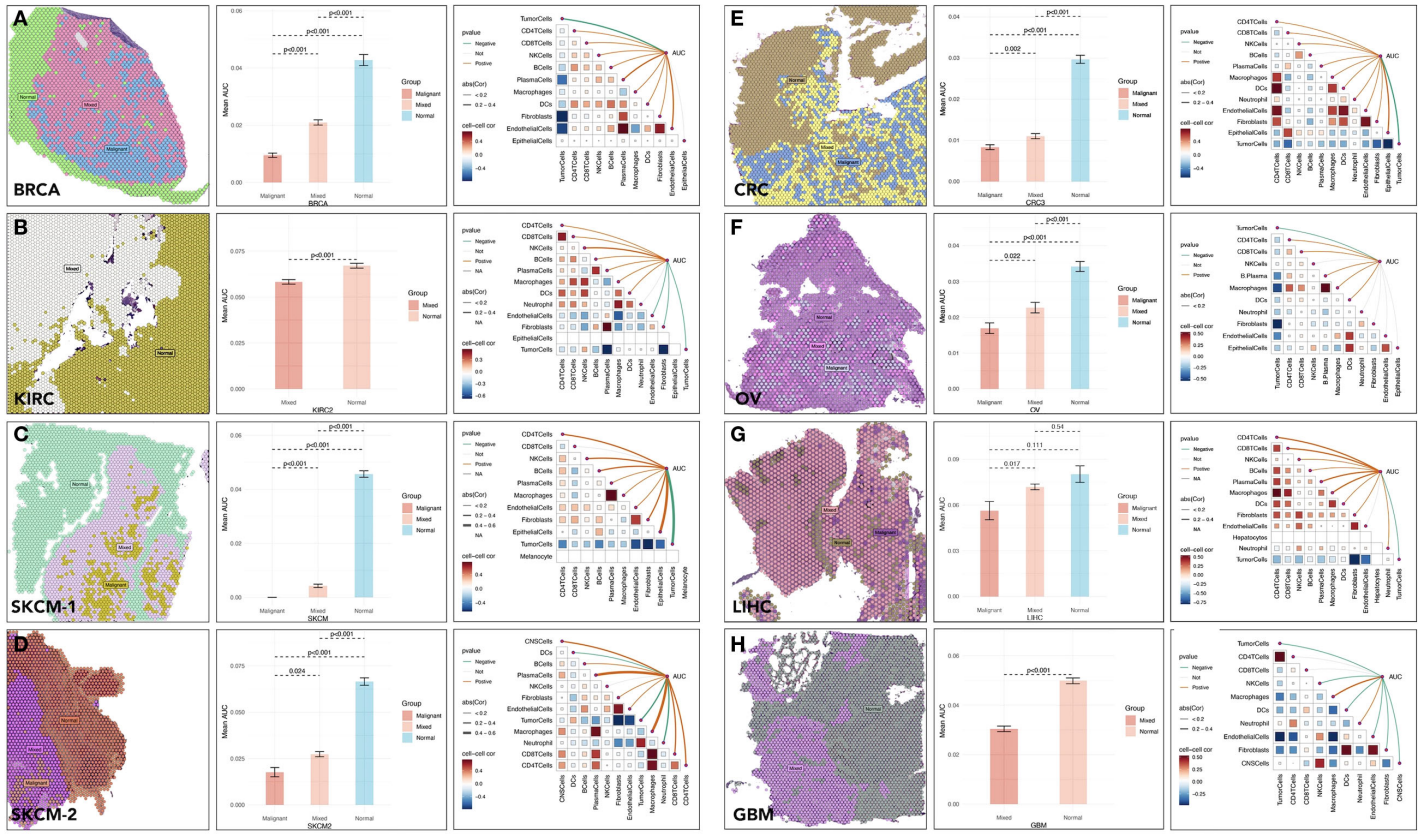
Spatial distribution and activity of specic gene sets in tumor microenvironments across cancer types. Spatial distribution of multi-gene AUC and immune cells correlation in **(A)** BRCA (Breast Cancer), **(B)** KIRC (Kidney Renal Clear Cell Carcinoma), **(C, D)** SKCM (Skin Cutaneous Melanoma), **(E)** CRC (Colorectal Cancer), **(F)** OV (Ovarian Cancer), **(G)** LIHC (Liver Hepatocellular Carcinoma), **(H)** GBM (Glioblastoma Multiforme).

Spatial transcriptomic analyses were conducted across several cancer types, including BRCA, KIRC, SKCM, and CRC. In BRCA, the expression of 11 pivotal genes, quantified by area under the curve(AUC) scores, showed a lower level in malignant tissues. For example, in BRCA, the mean AUC score was 0.097 in tumor tissues versus 0.043 in non-tumor tissues (p < 0.001) (**Figure 4A**). Besides, the AUC score had a negative correlation with tumor cell proportions (p < 0.001) (**Figure 4A**) and a positive correlation with CD8+ T cell infiltration (p < 0.001) (**Figure 4B**). Similar trends were observed in other types of cancer (**Figures 4C–H**). These results highlighted the role of these genes in shaping the tumor-immune microenvironment.

### 3.5 A refined grouping model for H-R and L-R group identification based on the 11 genes

**Figure 5:**
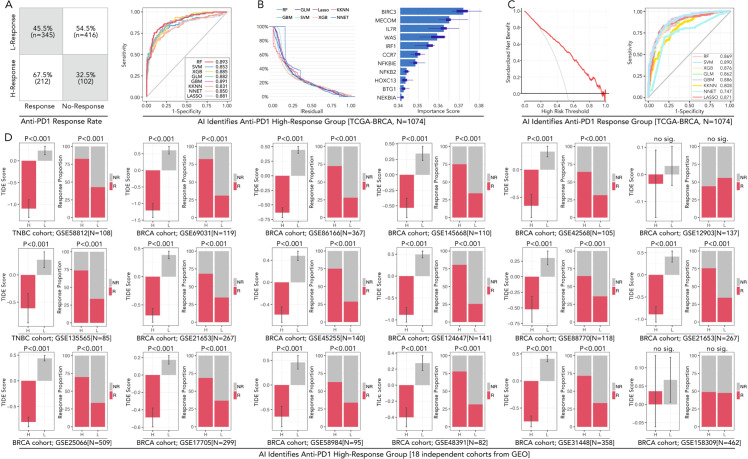
AI-powered prediction and validation of anti-PD1 high-response groups in breast cancer across TCGA and GEO cohorts. **(A)** The confusion matrix displays anti-PD1/PDL1 response rates in high-response (H-Response) and low-response (L-Response) groups identified in the TCGA-BRCA cohort (N=1074). **(B)** AUC performance of eight machine learning models in predicting immunotherapy subgroup, including RF (Random Forest), SVM (Support Vector Machine), XGB (XGBoost), KNN (k-Nearest Neighbors), LASSO, GBM (Gradient Boosting Machine), and NNET (Neural Network). Residual values of each algorithm. Variable importance scores for each gene, in which BIRC3 contributes most to the model prediction. **(C)** Decision curve analysis for evaluating the clinical utility of the predictive models and ROC curves shows SVM performed best in predicting anti-PD1/PDL1 response in BRCA. **(D)** Validation of AI-identified high-response groups across 18 independent BRCA cohorts from GEO datasets. Consistency about the accompanying event of the high-response (H-Response) group and higher antiPD1/PDL1 response is observed in 17/18 independent groups (except GES158309), the feature of which follows the TCGA cohort. In this part, Chi-square Test and Mann-Whitney U test were applied.

Machine learning models (eight types), including RF, GLM, LASSO, KKNN, GBM, SVM, XGB, NNET, were applied to predict high-response groups to anti-PD1 therapy. The RF model achieved the best performance, with an AUC of 0.893. GBM also demonstrated strong predictive capabilities, with AUC of 0.891. Decision curve analysis validated the clinical utility of these models, showing that RF consistently provided a good net benefit across a range of threshold probabilities (**Figures 5A–C**). Validation was conducted across 18 GEO cohorts comprising 3,425 samples. For example, in GSE12345, the high-response group identified by RF exhibited a response rate of 68%, compared to 35% in the low-response group (p < 0.001). In GSE54321, response rates were 72% and 38% in the high- and low-response groups, respectively (p < 0.001). Only one dataset (GSE158309) showed no significant difference in response rates (p = 0.14). These findings demonstrated the robustness and generalizability of the RF model in identifying immunotherapy responders across diverse datasets based on the 11 genes (**Figure 5D**).

### 3.6 Drug sensitivity prediction comparison by genome or multigene (11 genes)

**Figure 6:**
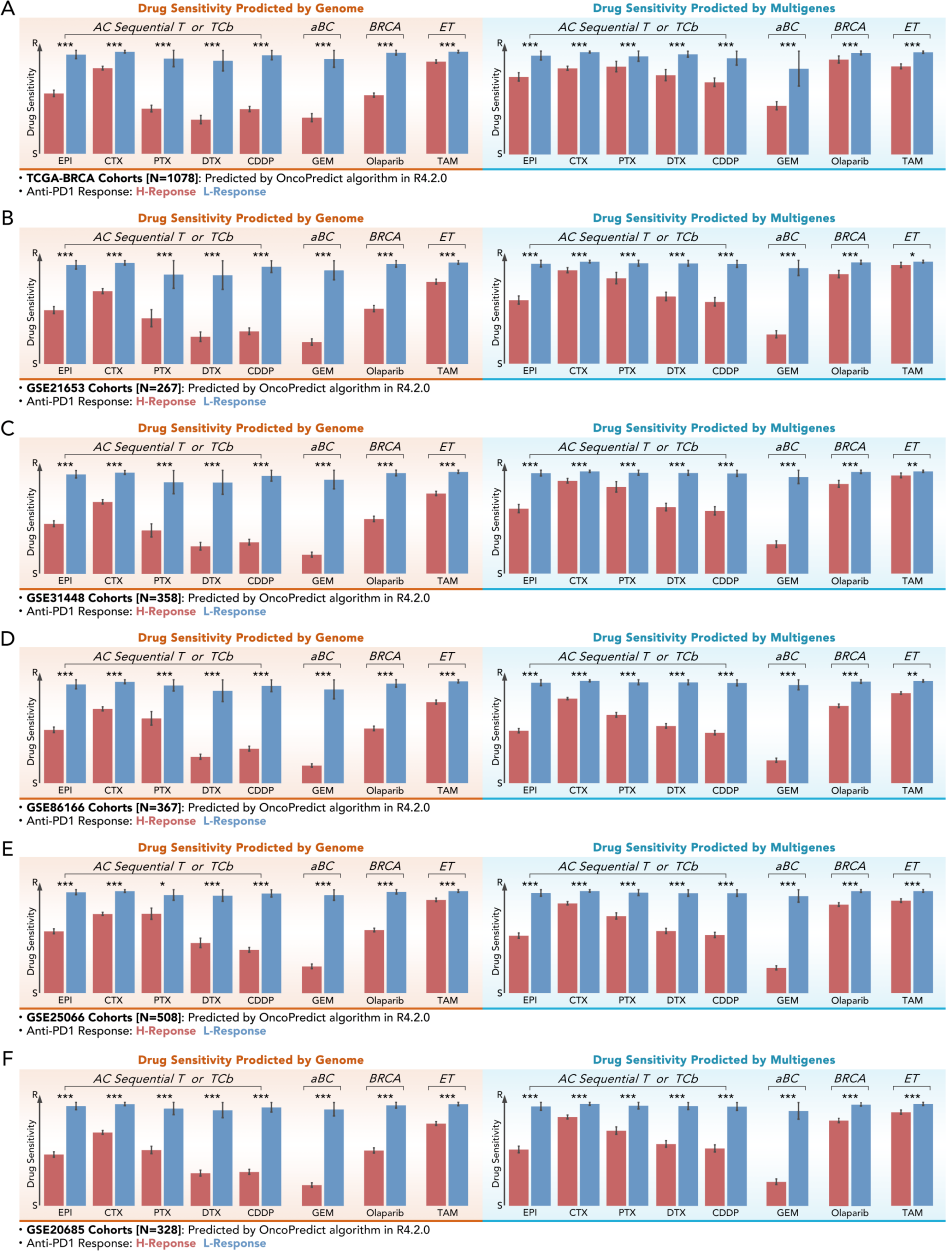
Multi-gene-based drug sensitivity prediction demonstrates comparable efficacy to genome-based prediction. **(A–F)** Drug sensitivity scores for different chemotherapy or endocrine agents (EPI, CTX, PTX, DTX, CDDP, GEM Olaparib, TAM) predicted by genome-based and multi-gene-based models in the help of “OncoPredict” algorithm across six independent cohorts: **(A)** TCGA-BRCA (N=1078), **(B)** GSE21653 (N=267), **(C)** GSE31448 (N=358), **(D)** GSE8166 (N=367), **(E)** GSE25066 (N=508), and **(F)** GSE20685 (N=328). All kinds of drug scores showed lower values in the high-response (H-Response) group with significance (Mann-Whitney U test, *p<0.05, **p<0.01, ***p<0.001).

The predictive performance of multi-gene models for drug sensitivity was assessed across six independent cohorts, including TCGA-BRCA (n=1078), GSE21653 (n=267), GSE31448 (n=358), GSE8166 (n=367), GSE25066 (n=508), and GSE20685 (n=328). Drug sensitivity scores were calculated for chemotherapeutic agents such as epirubicin (EPI), cyclophosphamide (CTX), paclitaxel (PTX), docetaxel (DTX), cisplatin (CDDP), gemcitabine (GEM), olaparib, and tamoxifen (TAM). In TCGA-BRCA, H-response groups exhibited significantly lower drug scores for chemotherapeutic drugs and endocrine compared to L-response groups (***p < 0.001), whatever in genome-based or multigene-based drug prediction (**Figures 6A–F**). Interestingly, those phenomena were verified in multiple independent GEO cohorts. These results underlined the potential of the 11-gene model to simplify the prediction of chemotherapy and endocrine therapy in BRCA.

### 3.7 Multi-gene prognostic model based on the MElightcyan module

**Figure 7:**
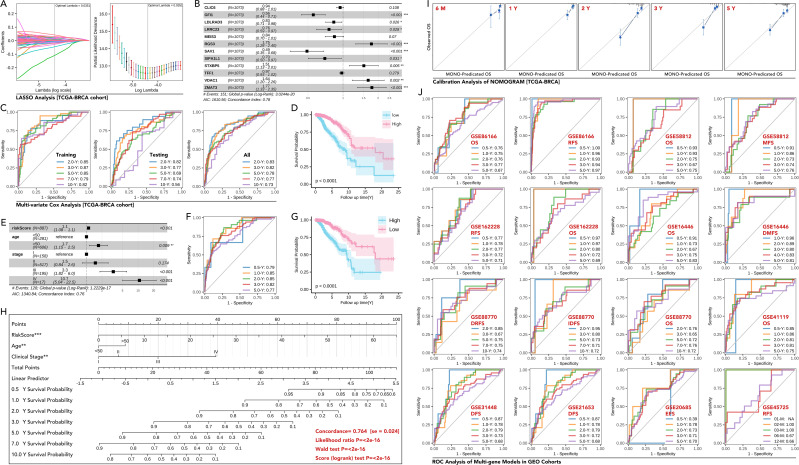
Development and validation of a multi-gene prognostic model based on MElightcyan module in breast cancer. **(A)** LASSO regression analysis to identify key genes from the MElightcyan module. The coefficient profiles of the genes, and the optimal lambda selected by 10-fold cross-validation. **(B)** Forest plot showing hazard ratios (HR) (univariate Cox regression) of the selected genes included in the multi-gene model (CLIC6, GFI1, LDLRAD3, LRRC23, MEIS3, RGS3, SAV1, SIPA1L1, STXBP5, TFF1, VDAC1, ZMAT3), in which GFI1, LDLRAD3, LRRC23, SAV1 and SIPA1L1 were prognosis-favorable genes (p<0.05), while RGS3, STXBP5, VDAC1 and ZMAT3 were risk factors for breast cancer prognosis (p<0.05). And those genes with statistical significance were input into further analysis. **(C)** ROC curves for the multi-gene prognostic model in the training, testing, and overall datasets of the TCGA-BRCA cohort, showing AUCs for predicting 0.5-, 2-, and 5-year overall survival (OS). **(D)** Kaplan-Meier survival curves demonstrating significant OS differences between the high-risk and low-risk groups stratified by the multi-gene score (Higher risk Score with better prognosis). **(E)** Multivariate Cox regression analysis of the risk score and clinical features (age, stage), showing the risk score as an independent prognostic factor (p<0.0001). **(F, G)** Kaplan-Meier curves and ROC analysis of Multivariate Cox regression analysis. **(H)** Nomogram for predicting 1-, 3-, and 5-year survival probabilities in breast cancer patients based on the risk score, age, and clinical stage, concordance of which is equal to 0.764 (se=0.024) with statistical significance (p<0.0001). **(I)** Calibration plots comparing predicted and observed OS at different time points (6 months, 1 year, 2 years, 3 years, 5 years) in the TCGA-BRCA cohort. **(J)** ROC curves for OS, RFS (recurrence-free survival), DFS (disease-free survival), and DMFS (distant metastasis-free survival) in various independent GEO datasets (GSE8166, GSE58812, GSE162228, GSE16446, GSE88770, GSE41119, GSE31448, GSE21653, GSE20685 and GSE45725). AUC values demonstrate robust prognostic performance across datasets. * p<0.05, ** p<0.01, ***p<0.001.

The MElightcyan module gene set (**Figure 3B**) was refined through LASSO analysis, and subsequently subjected to univariate Cox regression. This process resulted in the identification of nine genes: GFI1, LDLRAD3, LRRC23, RGS3, SAV1, SIPA1L1, STXBP5, VDAC1, and ZMAT3) (**Figures 7A, B**). The 9-gene prognostic model by multivariate Cox regression based on the TCGA-BRCA training cohort was tested in the testing TCGA-BRCA cohort (70% was training cohort, 30% was testing cohort), with AUC values of 0.83, 0.82, 0.78, 0.77, and 0.73 for predicting 0.5-, 2-, and 5-year OS in all cohort, respectively (**Figure 7C**). Then, Kaplan-Meier survival analysis demonstrated a significant difference in overall survival (OS) between high-risk and low-risk groups (50% vs 50% calculated by the model) (p<0.0001, **Figure 7D**). Then the 11-gene riskScore and clinical characteristics were put into multivariate Cox regression, which selected riskScore, age, and stage as parameters (**Figure 7E**). The new model’s predictive accuracy was validated through ROC curve analysis, with AUC values of 0.79, 0.85, 0.85, 0.82, and 0.77 for predicting 0.5-, 1-, 2-, 3-, and 5-year OS, respectively (**Figure 7F**). Kaplan-Meier survival analysis demonstrated a significant difference in overall survival (OS) between high-risk and low-risk groups (50% vs 50% calculated by the new model) (p<0.0001, **Figure 7G**). The new multivariate Cox regression model was shown as a nomogram (**Figure 7H**), and the prognosis prediction efficacy was evaluated by calibration (**Figure 7I**). Validation in external GEO cohorts, such as GSE25066, confirmed the robustness of the model (**Figure 7J**). These findings highlighted the utility of the 9-gene signature as a robust prognostic tool.

### 3.8 miR-26a-5p/HOXC13 as an immunotherapy regulating pathway

**Figure 8:**
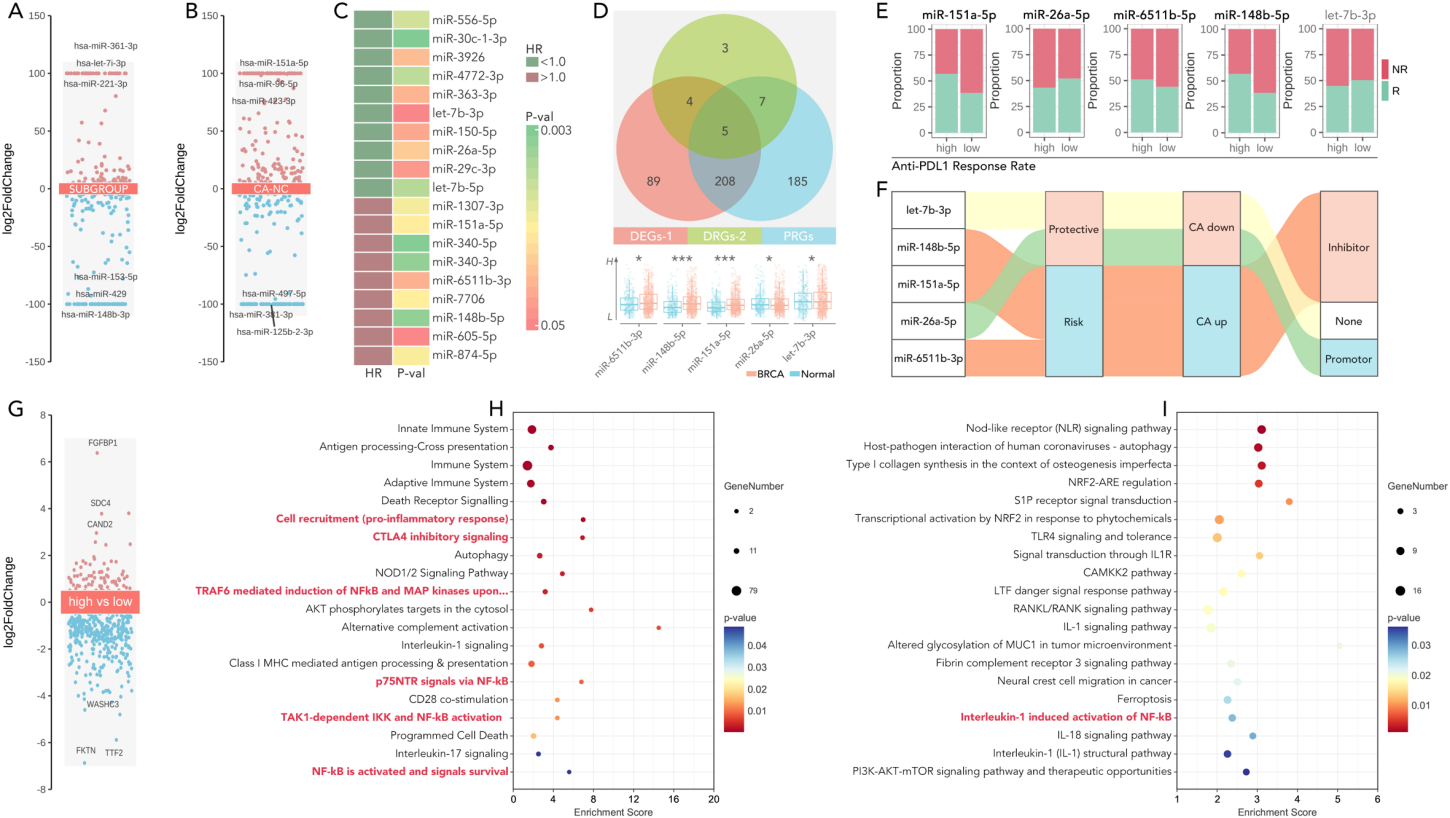
miR-26a-5p is a potential immunotherapy regulator in the cGAS-STING-based multi-gene risk model. **(A)** Volcano plots showing differentially expressed miRNAs (DEGs) in breast cancer across subgroups, calculated by limma package in R. (*limma* package, threshold: p<0.05, log|FC|>=1). **(B)** Volcano plots showing differentially expressed miRNAs (DEGs) in breast cancer between CA (breast cancer) and NC (normal), calculated by limma package in R. (*limma* package, threshold: p<0.05, log|FC|>=1). **(C)** Heatmap of significant miRNAs stratified by hazard ratio (HR <1.0 indicates favorable prognosis) and corresponding P-values. **(D)** Overlapping miRNAs between three groups (differentially expressed miRNA (DEGs-1), drug differentially expressed miRNA (DEGs-2), and prognosis-related miRNA (PRGs)), and miR-65611b-5p, miR-148b-5p, miR-151a-5p, miR-26a-5p and let-7b-3p were identified as candidates for further analysis. (*p<0.05, **P<0.01, ***p<0.001). **(E)** Proportion of anti-PD1/PDL1 responders (R) and non-responders (NR) in high- and low-expression groups of miRNAs, which showed the higher level of miR-151a-5p, miR-6511b-5p and miR-148b-5p held higher response rate, while higher level of miR-26a-5p held lower response rate (Chi-square Test, p<0.05), no significance was observed in let-7b-3p. **(F)** Sankey diagram illustrating the roles of selected miRNAs in cancer, categorized as protective, risk-related, inhibitor, or promoter miRNAs, based on their functional annotations. **(G)** Volcano plot of differentially expressed genes between the high-expression group and low-expression group, divided by the level of miR-26a-5p. (*limma* package, threshold: p<0.05, log|FC|>=1). **(H, I)** Pathways enrichment analysis (KEGG and wiki pathway enrichment) of differently expressed genes in **(G)**, which showed NF-kB activation, IL-1 signaling, and PI3K-AKT-mTOR pathways, etc. were significant.

Differentially expressed miRNAs across subgroups (H-response/L-response) (**Figure 8A**) and different tissues (breast cancer tissues/normal tissues) (**Figure 8B**) were identified. The prognosis-related miRNAs were also identified (**Figure 8C**). Five shared miRNAs were finally filtered (miR-6511b-3p, miR148b-5p, miR151a-3p, miR-26a-5p, let-7b-3p), amongst which miR-26a-5p was lower expressed in breast cancer, while other four miRNAs were higher expressed (**Figure 8D**). Following, differences in predicted anti-PDL1/PD1 response rate were calculated in the high-expression and low-expression groups of the 5 miRNAs, amongst which the high-expression group of two miRNAs held a lower response rate, while the other three miRNAs exhibited opposite performance (**Figure 8E**). Sankey diagram showed an interesting result, that the higher expressed of miRNA, reckoned as potential carcinogenetic factors, was accompanied by higher anti-PD1/PDL1 rate, while only anti-cancer miR-26a-5p performed immunotherapy-resistance effect (**Figure 8F**). So, miR-26a-5p was put into further analysis. Differently expressed genes were filtered between the high-expressed miR-26a-5p group and the low-expressed miR-26a-5p group (**Figure 8G**). KEGG and Wikipathway enrichment analysis was performed, in which the former analysis revealed that high miR-26a-5p expression activated immune suppression pathways, including NF-κB signaling and CTLA4 inhibitory signaling (**Figure 8H**), and the later analysis also identified NF-κB signaling (**Figure 8I**). These findings highlight the role of miR-26a-5p as a regulator of immune evasion and a potential biomarker for immunotherapy resistance.

**Figure 9:**
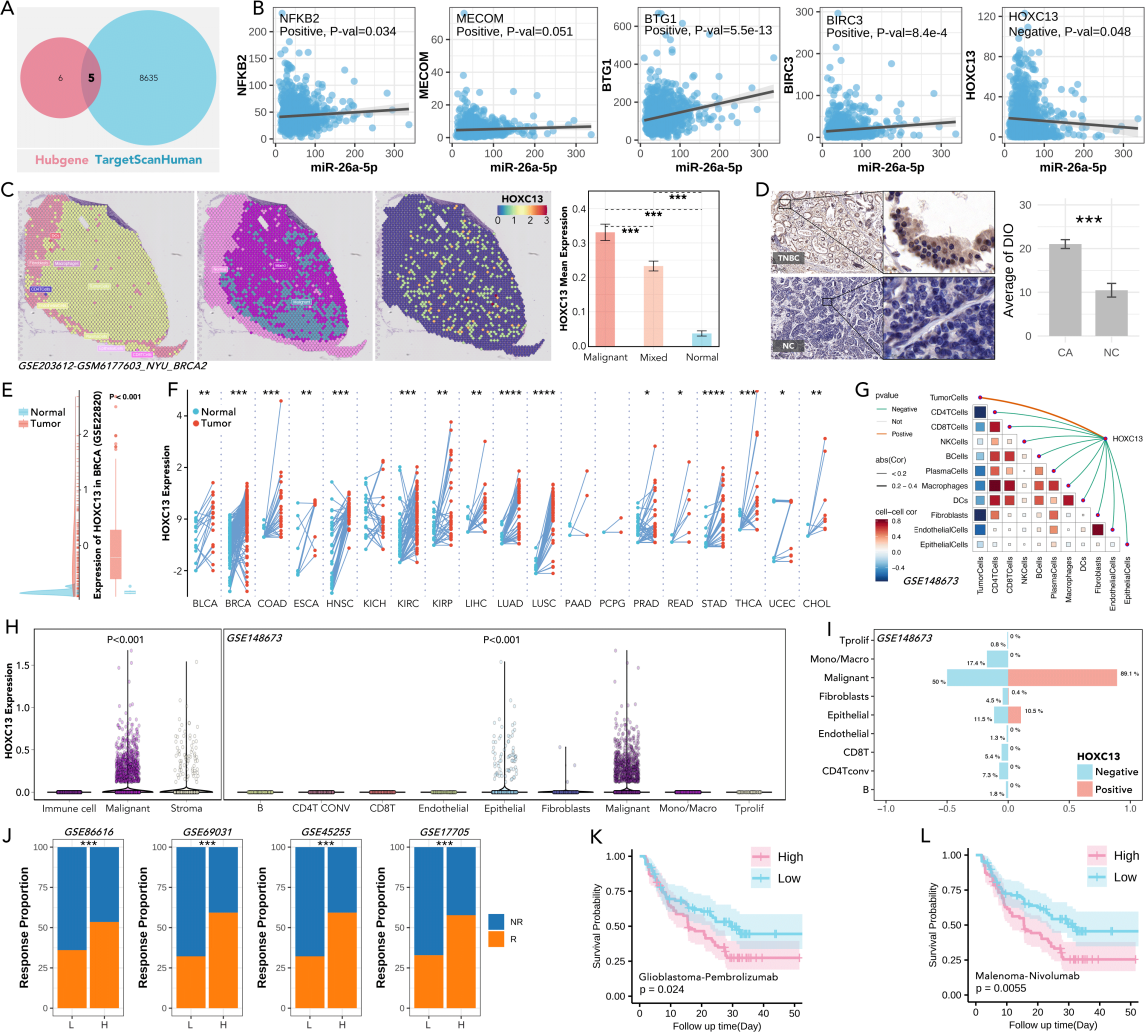
Role of HOXC13 in tumor biology, immune modulation, and response to immunotherapy. **(A)** Shared targets of miR-26a-5p targets predicted from TargetScanHuman and Hubgene databases. **(B)** Correlation analysis between miR-26a-5p expression and key target genes, including NFKB2, MECOM, BTG1, BIRC3, and HOXC13, in which HOXC13 is negatively regulated by miR-26a-5p. (simple linear regression). **(C)** Spatial transcriptomic expression feature of HOXC13 in breast cancer, which showed the highest expression level in breast cancer tissues (Mann-Whitney U test, ***p<0.001). Data from GSE203612. **(D)** IHC staining of HOXC13 in a local cohort, which showed triple-negative breast cancer (TNBC) tissues held higher expression levels (Paired Sample t Teat, ***p<0.001). **(E)** HOXC13 expression in the GEO (GSE22820) cohort, showing significant upregulation in tumor versus normal tissues (Mann-Whitney U test, P<0.001). **(F)** HOXC13 expression across pan-cancer from TCGA, which reveals consistent overexpression in tumor tissues (Mann-Whitney U test, ***p<0.001). **(G)** Correlation between HOXC13 expression and cells, which displayed a positive correlation between HOXC13 and tumor cells, and a negative correlation between HOXC13 and other immune cells. Data from GSE148673. **(H)** HOXC13 expression in immune, malignant, stromal, and epithelial cells, with significantly higher expression in malignant cells (Mann-Whitney U test, P<0.001). Data from GSE148673. **(I)** Immune cells and tumor cells infiltration proportion between the higher expression group of HOXC13 and low expression group, which showed a higher level of HOXC13 accompanied by a higher level of malignant cell, but a lower level of CD8 T cells. Data from GSE148673. Chi-square Test. **(J)** Anti-PD1/PLD1 response proportions in cohorts stratified by HOXC13 expression. High HOXC13 expression correlates with a higher response rate across multiple datasets. Data from GSE86616, GSE69031, GSE45255, and GSE17705. (Chi-square Test). **(K)** Higher HOXC13 expression exhibited significantly worse outcomes in glioblastoma treated with pembrolizumab (log rank p=0.024). **(L)** Higher HOXC13 expression exhibited significantly worse outcomes in melanoma treated with nivolumab (log rank p=0.0055). * p<0.05, ** p<0.01, ***p<0.001, ****p<0.0001.

Following, five shared genes were filtered across hub-genes and TargetScanHuman database (**Figure 9A**), amongst which HOXC13 was negatively correlative with miR-26a-5p (p=0.048, **Figure 9B**). Spatial expression of HOXC13 was highest in the malignant area as compared with normal or mixed area (***: p<0.001, **Figure 9C**). The phenomenon of higher expression in triple-negative breast cancer tissues was observed in a local cohort (p<0.001, **Figure 9D**), which was also verified in the GEP cohort (GSE2820, p<0.001, **Figure 9E**). Furthermore, pan-cancer analysis showed HOXC13 was highly expressed in 17 types of cancer (**Figure 9F**). Spatial transcription analysis showed HOXC13 was positively correlated with tumor cell proportions (r = 0.68, p < 0.01) and negatively with CD8+ T cell infiltration (r = -0.54, p < 0.01) (**Figure 9G**). Besides, HOXC13 expression in the microenvironment was also explored, and it displayed the same result that malignant tissues held the highest expression (p<0.001, GSE148673, **Figure 9H**). In microenvironment gradient analysis, higher expressed HOXC13 was accompanied by a higher rate of malignant cells (positive vs negative: 89.1% vs 50%), while a lower rate of CD8 T cells was observed (positive vs negative: 5.4% vs 0%) (**Figure 9I**). Finally, the immunotherapeutic effect of HOXC13 in breast cancer was explored, and results showed that the group with higher expressed HOXC13 held higher anti-PD1/PDL1 response rates in four independent cohorts (**Figure 9J**). In real-world studies, in the glioblastoma and melanoma cohort, the group with higher levels of HOXC13 displayed a worse prognosis after immunotherapy (**Figures 9K, L**). Besides, clinical parameters (such as age, N stage, etc.) also explored in HOXC13-regulated anti-PD1/PDL1 response and prognosis (**Supplementary Figure S3**). Those results implied that HOXC13 is a potential target for enhanced immunotherapy which is probably regulated by miR-26a-5p.

**Figure 10:**
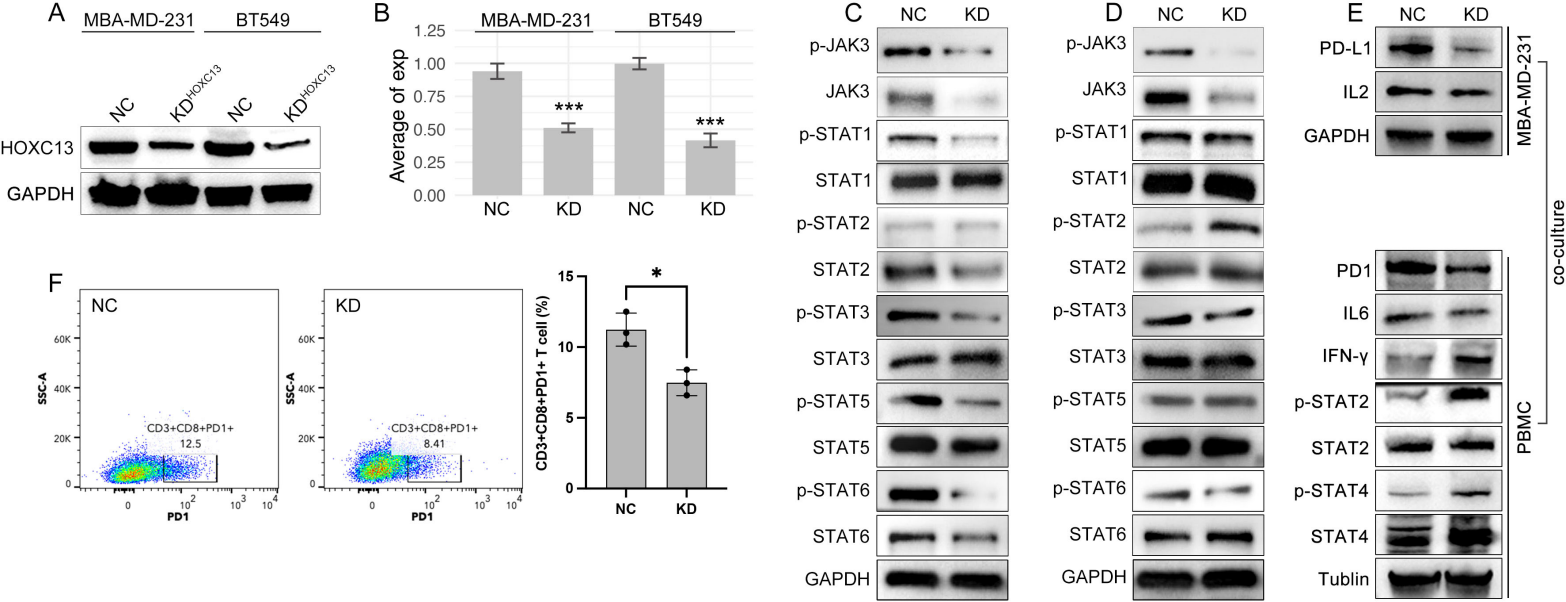
HOXC13 knockdown modulates JAK-STAT/IL6 signaling and T cell exhaustion. **(A)** Western blot analysis confirms reduced HOXC13 expression in HOXC13-KD cells (MBA-MD-231, BT549). **(B)** Quantification of HOXC13 expression levels (normalized to GAPDH), showing significant knockdown efficiency (Mann-Whitney U test, ***P<0.001). **(C, D)** Decreased phosphorylation of JAK3, STAT1, STAT3, STAT5, and STAT6 in HOXC13-KD MBA-MD-231 or BT-549 cells compared to NC, while phosphorylation of STAT2 is increased only in HOXC13-KD BT549 cells. **(E)** Co-culture of HOXC13-KD MBA-MD-231 cells and PBMCs, in which HOXC13-KD decreased expression of PD-L1 and IL2, and the further effect is downregulation of PD1 and IL6, and the up-regulation of IFN-gamma, phosphorylated STAT2/4. **(F)** Flow cytometry analysis of T cell function in PBMCs co-cultured with NC or HOXC13-KD MBA-MD-231 cells, which displayed that PD1+CD3+CD8+ T proportion is decreased in HOXC13-KD group (NC: 12.5% vs HOXC13-KD 8.41%). * means p<0.05.

### 3.9 miR-26a-5p/HOXC13 pathway regulated proliferation, migration, and drug resistance

**Figure 11:**
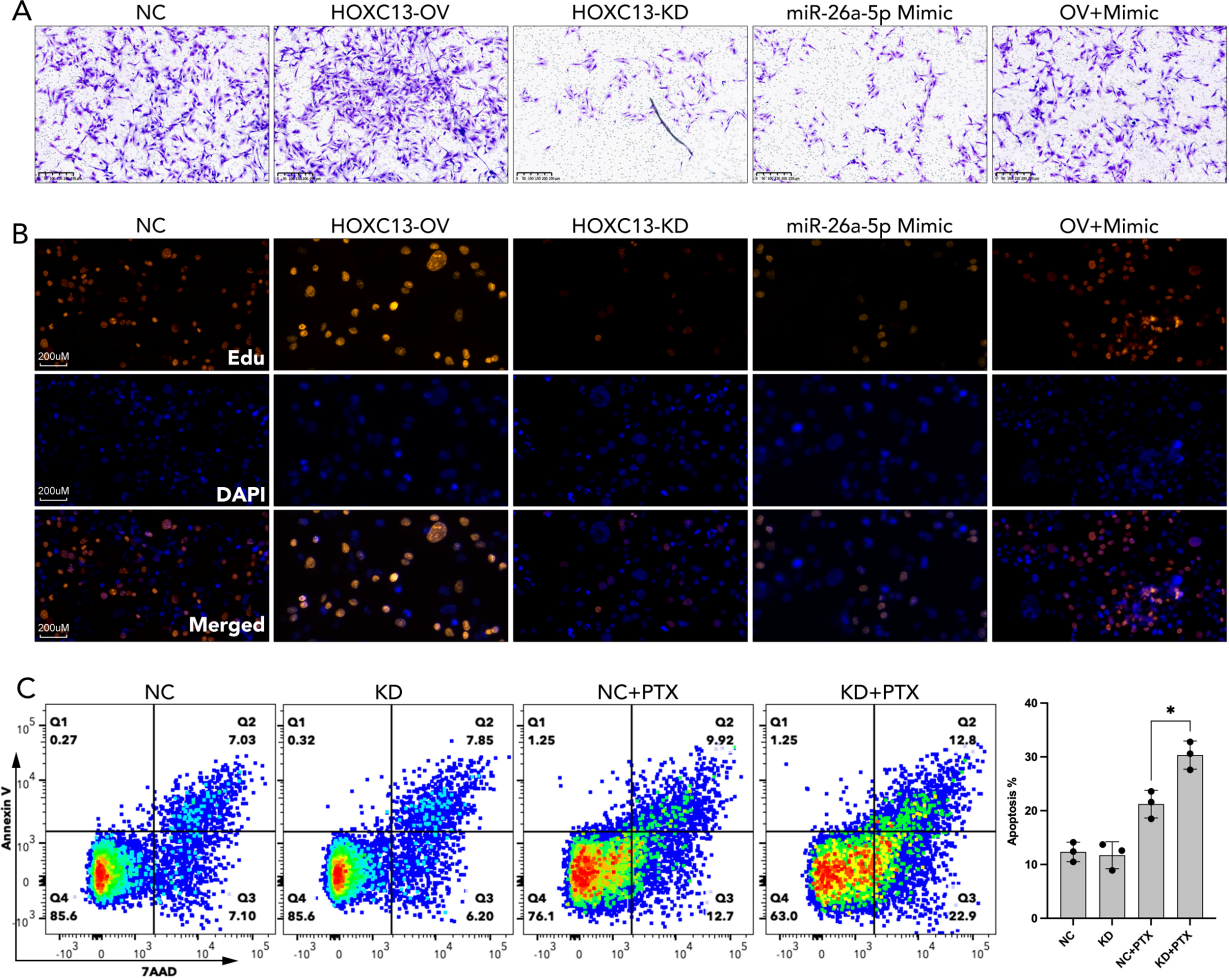
miR-26a-5p/HOXC13 pathway regulated cell migration, proliferation, and apoptosis in breast cancer. **(A)** Trans-well assay demonstrated the larger amount of migrated breast cancer cells (MBA-MD-231) in the HOXC13-OV group while a smaller amount of migrated cells is observed in the HOXC13-KD group and miR-26a-5p mimic group as compared to NC group. NC: Negative control cells. HOXC13-OV: HOXC13 overexpression significantly enhances migration. HOXC13-KD: HOXC13 knockdown reduces migration. OV+Mimic: Co-treatment with HOXC13 overexpression and miR-26a-5p mimic. **(B)** EdU assay showed stronger cell proliferation in the HOXC13-OV group while weaker cell proliferation was observed in the HOXC13-KD group and miR-26a-5p mimic group as compared to the NC group. Edu (Red): Proliferating cells. DAPI (Blue): Nuclear staining. Merged: The combined image of EdU and DAPI. **(C)** Flow cytometric analysis of apoptosis in breast cancer cells treated with paclitaxel (PTX). NC, Negative control; KD, HOXC13 knockdown. * p<0.05.

Functional assays demonstrated that HOXC13 over-expression elevated migrated cellular area by ~20% and ~50% Edu staining strength, which were partially rescued by miR-26a-5p mimic (**Figures 11A, B**). On the contrary, the knockdown of HOXC13 significantly inhibited the cellular migration and proliferation (Edu staining strength), which was similar to the performance of miR-26a-5p mimic (**Figures 11A, B**). Following, chemotherapy resistance was explored, and flow cytometric analysis revealed that HOXC13 knockdown increased apoptosis by 36.6% in paclitaxel-treated cells, while a single treatment of HOXC13 knockdown did few effects on cell apoptosis (NC vs KD=14.13% vs 14.05%) (**Figure 11C**).

### 3.10 HOXC13 regulated CD8 T cell exhaustion by JAK-STAT/ILs pathway

**Figure 12:**
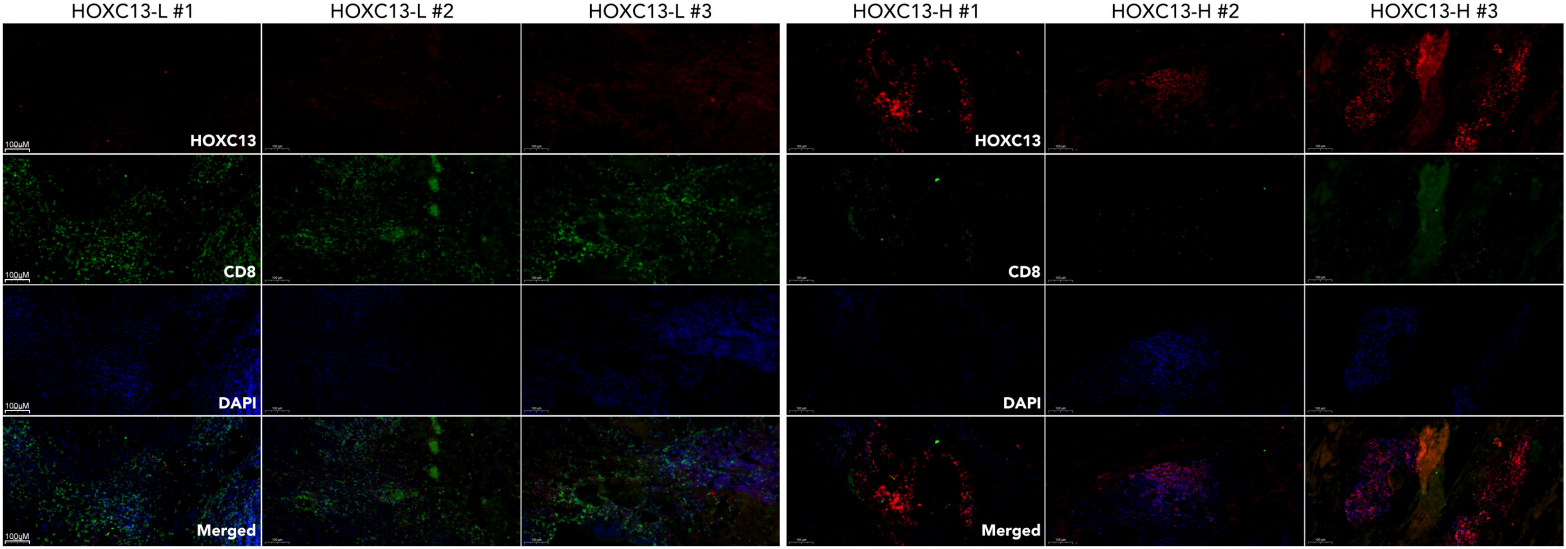
Differential expression of HOXC13 and CD8+ T cell infiltration in breast cancer tissues. Immunofluorescence (IF) staining of breast cancer tissues with high (HOXC13-H) and low (HOXC13-L) HOXC13 expression levels. HOXC13 (Red): HOXC13 expression is significantly higher in HOXC13-H tissues compared to HOXC13-L tissues. CD8 (Green): CD8+ T cell infiltration is more prominent in HOXC13-L tissues, indicating a potential inverse correlation between HOXC13 expression and CD8+ T cell presence. DAPI (Blue): Nuclear staining.

To further explore the mechanisms of that HOXC13 regulated immune escape, HOXC13 expression was downregulated by small RNA (siRNA) in TNBC cell lines (MBA-MD-231 and BT549) (**Figures 10A, B**, ***p<0.001). The downregulation of HOXC13 led to the decrease of phosphorylated JAK3 (p-JAK3), p-STAT1, p-STAT3, p-STAT5, p-STAT6, and the increase of p-STAT2 (**Figures 10C, D**). Furthermore, PBMCs were extracted and co-cultured with MBA-MD-231. Western blot (WB) assay showed the HOXC13 knockdown decreased the level of PD-L1, and also led to the decrease of PD1 and IL6 in PBMC, while INF-gamma, p-STAT2, and p-STAT4 were increased in PBMC (**Figure 10E**). Besides, FACS was performed and results showed that HOXC13 downregulation decreased the proportion of PD1+CD3+CD8+ cells (**Figure 10F**). Following, the correlation between HOXC13 and CD8+ T cell infiltration was explored, which displayed that a higher expression level of HOXC13 was accompanied by the lower infiltrated CD8+ cells in TNBC (**Figure 12**).

## 4 Discussion

Our study highlights the critical role of the cGAS-STING pathway, molecular subtypes, and the miR-26a-5p/HOXC13 axis in breast cancer immune modulation and therapy response. These findings not only expand our understanding of the tumor microenvironment but also identify potential biomarkers and therapeutic targets that can guide personalized treatment strategies.

The cGAS-STING pathway is traditionally recognized as a key driver of innate immune responses, promoting antitumor immunity through the activation of type I interferons (25–27). However, our results suggested that the overexpression of cGAS-STING pathway genes was paradoxically associated with poor outcomes in breast cancer, particularly by promoting immune evasion. We observed that higher expression levels of genes like DTX4 and STING were correlated with increased proportions of non-responders to anti-PD1 therapy and elevated TIDE scores, indicating greater immune dysfunction. These findings align with recent studies suggesting that chronic activation of the STING pathway can lead to immune exhaustion and a tolerogenic microenvironment, potentially driven by sustained production of immunosuppressive cytokines or recruitment of regulatory immune cells (28–30). Thus, while the cGAS-STING pathway holds promise as a target for immunotherapy, its dual roles in antitumor immunity and immune suppression must be carefully balanced. Future investigations should focus on the contextual factors—such as timing, dose, and tumor subtype—that influence the pathway’s effects on immune responses.

Our molecular subtyping analysis underscored the heterogeneity of breast cancer and its profound implications for immunotherapy. Subtype C4, identified through consensus clustering, exhibited the most immune-excluded phenotype, with the highest TIDE scores, elevated immune checkpoint expression, and poor survival outcomes. This subtype was also characterized by enhanced expression of immune suppressive markers such as PD1, PDL1, and CTLA4, supporting its immune-resistant profile. These findings are consistent with previous reports that specific breast cancer subtypes, particularly those with mesenchymal or basal-like features, were more prone to immune evasion (31–34). On the other hand, subtype C7, identified through non-negative matrix factorization, showed higher proportions of responders and lower TIDE scores, suggesting a more immunogenic environment. Importantly, survival analysis revealed that subtypes defined by consensus clustering were more predictive of clinical outcomes than those identified by NMF. This highlights the importance of using robust, biologically informed methods to classify tumors and guide therapy decisions. Further studies are needed to validate these subtypes across larger, independent cohorts and to investigate their underlying biology.

The miR-26a-5p/HOXC13 axis represents a newly identified pathway with significant implications for immunotherapy response and tumor progression. Our findings showed that miR-26a-5p is downregulated in breast cancer and is associated with immune suppression through pathways like NF-κB signaling and CTLA4 inhibition. Interestingly, miR-26a-5p was found to negatively regulate HOXC13, a transcription factor whose expression was highest in triple-negative breast cancer and associated with poor immunotherapy response. The pan-cancer analysis further demonstrated that HOXC13 is upregulated in multiple tumor types, and its expression correlates with increased tumor cell proportions and decreased CD8+ T cell infiltration. Functional assays revealed that HOXC13 overexpression promoted proliferation, migration, and chemotherapy resistance, while its knockdown restored sensitivity to paclitaxel and enhanced immune cell infiltration. Mechanistically, HOXC13 appears to drive immune escape through the JAK-STAT pathway, altering the expression of immune checkpoint molecules and interleukins. These results are consistent with previous studies linking HOX family genes to cancer progression and immune modulation (35–41). miR-26a-5p has also been reported as an oncogene in a variety of cancers (42–47). Our study further explored this area by identifying miR-26a-5p as a potential upstream regulator and by demonstrating the pathway’s relevance to both immune escape and drug resistance.

Our study combines bioinformatics and experimental validation to pinpoint novel biomarkers and therapeutic targets for breast cancer, focusing on high-risk subtypes like TNBC. We identified subtype-specific immune landscapes and the miR-26a-5p/HOXC13 axis as critical immune response regulators. Patients with elevated HOXC13 or immune-excluded subtypes may respond poorly to checkpoint inhibitors alone, suggesting combination therapies targeting the JAK-STAT pathway, HOXC13, or miR-26a-5p restoration. Additionally, machine learning models enhance our ability to predict immunotherapy responses, advancing precision oncology by enabling better patient stratification and treatment planning.

However, limitations temper our findings. The retrospective analysis requires prospective clinical studies to validate these biomarkers and subtypes. While the miR-26a-5p/HOXC13 axis showed promise *in vitro*, *in vivo* studies are essential to confirm its therapeutic potential and immune interactions. Relying on TIDE scores for immune dysfunction assessment calls for broader profiling with markers like tumor mutational burden and spatial immune data. The cGAS-STING pathway’s dual role in immunity also needs further exploration. Despite these challenges, our work lays a groundwork for personalized immunotherapies, aiming to boost treatment success for breast cancer patients.

## 5 Conclusion

Our study implies that HOXC13 is a potential therapy target for BRCA immunotherapy and 11-gene signature is a potential tool for clinical evaluation of anti-PD1/PDL1 therapy efficacy. The miR-26a-5p/HOXC13 axis is pivotal in shaping breast cancer immunity and therapy response. These findings offer valuable insights into the mechanisms of immune evasion and resistance to breast cancer.

## Data Availability

The datasets presented in this study can be found in online repositories. The names of the repository/repositories and accession number(s) can be found in the article/**Supplementary Material**.

## Supplementary material

Supplementary Figure 1 Functional enrichment analysis of differentially expressed genes across molecular subtypes in breast cancer. **(A)** Volcano plot showing the distribution of differentially expressed genes (DEGs) across molecular subtypes (C1, C2, C3, C4). Genes with significant changes in expression (log2 fold change > 1 or < -1, P < 0.05) are highlighted for each subtype. **(B)** Venn diagram illustrating the overlap of DEGs among different subtypes. **(C, D)** Pathway analysis about 161 overlapped genes across subtypes (C1, C2, C3, C4).

Supplementary Figure 2 Meta-analysis of hazard ratios for the association between biomarker expression and survival outcomes across multiple breast cancer cohorts. Forest plot summarizing the meta-analysis of hazard ratios (HR) for the association between HOXC13 expression and survival outcomes across multiple breast cancer datasets. The pooled hazard ratio is represented by the red diamond at the bottom, indicating a significant association (HR = 1.13, 95% CI [1.07–1.19], P < 0.05). The heterogeneity across studies is assessed with I² = 36%, τ² = 0.0057, and P = 0.03, suggesting moderate heterogeneity.
