# Supplementary figures and images for: An immunotherapy guide constructed by cGAS-STING signature for breast cancer and the biofunction validation of the pivotal gene HOXC13 via in vitro experiments
Source: Front Immunol. 2025 Aug 8;16:1586877. doi: 10.3389/fimmu.2025.1586877 (PMC12370726; doi:10.3389/fimmu.2025.1586877)

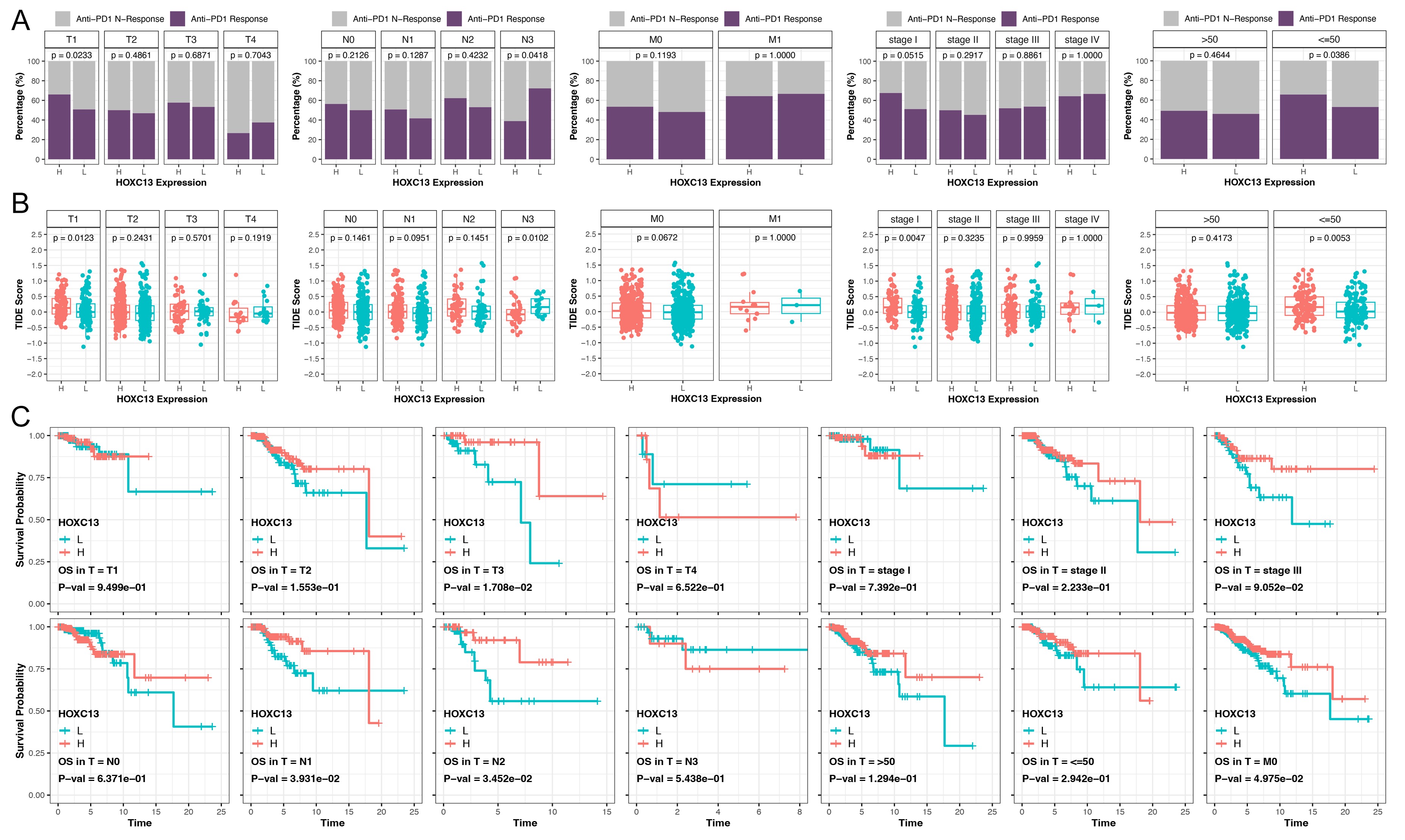

Supplement: Supplementary Figure 1 — Functional enrichment analysis of differentially expressed genes across molecular subtypes in breast cancer. (A) Volcano plot showing the distribution of differentially expressed genes (DEGs) across molecular subtypes (C1, C2, C3, C4). Genes with significant changes in expression (log2 fold change > 1 or < -1, P < 0.05) are highlighted for each subtype. (B) Venn diagram illustrating the overlap of DEGs among different subtypes. (C, D) Pathway analysis about 161 overlapped genes across subtypes (C1, C2, C3, C4). [file Image1.tif]

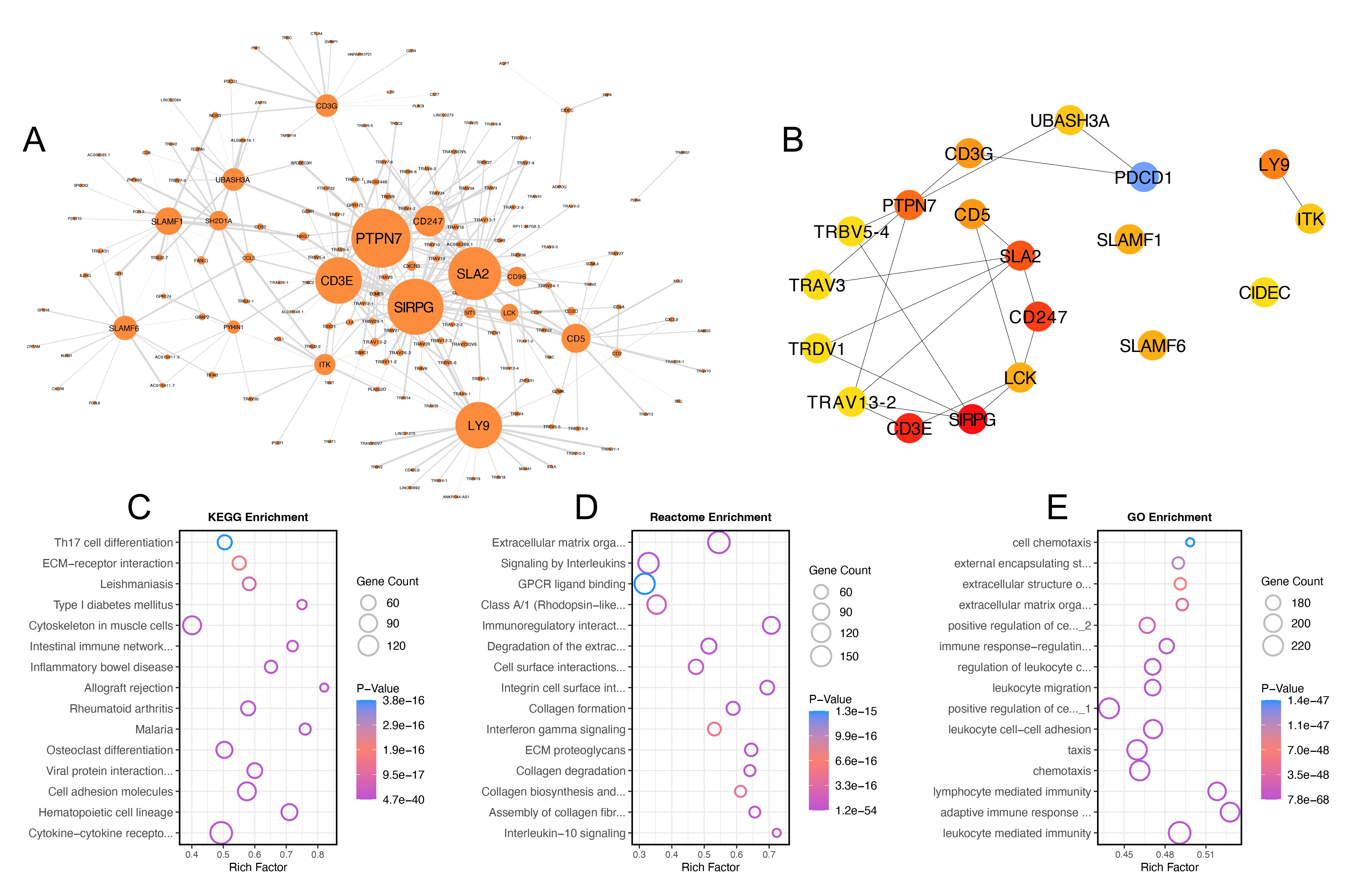

Supplement: Supplementary Figure 2 — Meta-analysis of hazard ratios for the association between biomarker expression and survival outcomes across multiple breast cancer cohorts. Forest plot summarizing the meta-analysis of hazard ratios (HR) for the association between HOXC13 expression and survival outcomes across multiple breast cancer datasets. The pooled hazard ratio is represented by the red diamond at the bottom, indicating a significant association (HR = 1.13, 95% CI [1.07–1.19], P < 0.05). The heterogeneity across studies is assessed with I² = 36%, τ² = 0.0057, and P = 0.03, suggesting moderate heterogeneity. [file Image2.tif]
