# Supplementary material for: An immunotherapy guide constructed by cGAS-STING signature for breast cancer and the biofunction validation of the pivotal gene HOXC13 via in vitro experiments
Source: Front Immunol. 2025 Aug 8;16:1586877. doi: 10.3389/fimmu.2025.1586877 (PMC12370726; doi:10.3389/fimmu.2025.1586877)
Supplement: Supplementary file 3 [file Table1.docx]

**Dear Responsible Editor Prof. Ming Xu**

**We are very grateful for the valuable comments from reviewers, we have finished modification of the manuscript “C An immunotherapy guide constructed by cGAS-STING signature for breast cancer and the biofunction validation of the pivotal gene HOXC13 via in vitro experiment”. Following were details:**

***PART-1 Review Comments***

1.Further elaborate on the current status of the cGAS-STING pathway in the field of breast cancer immunotherapy research, emphasizing the gaps filled by this study.
2.Provide detailed parameter settings of each machine-learning algorithm to enhance the reproducibility of the methods.
3.In the cell experiment section, supplement details such as the number of cell culture passages and the detection of transfection efficiency.
4.When presenting the relationship between gene expression and immunotherapy response, increase the analysis of more clinical feature data.
5.In the weighted gene co - expression network analysis (WGCNA), in addition to identifying gene modules related to immune response and subgroup classification, further explore the interaction mechanisms of genes within the module. For example, construct a gene regulatory network to clarify the upstream and downstream relationships between core genes and other genes, and excavate potential regulatory pathways, providing a more comprehensive perspective for understanding the molecular mechanism of breast cancer immunotherapy.
6.Currently, the research mainly relies on in vitro experiments to verify the function of HOXC13. In-vivo animal experiments should be carried out. Establish a breast cancer mouse model, and by knocking down or overexpressing the HOXC13 gene, observe tumor growth, immune cell infiltration, and the response to immunotherapy drugs to verify its role in the in - vivo environment and enhance the clinical translation value of the research results.
7.This research is mainly based on transcriptome data. Proteomics, metabolomics, and other multi - omics data can be integrated. Proteomics data can directly reflect the final products of gene expression, and metabolomics data can reveal cell metabolic changes. Multi-omics joint analysis helps to more deeply understand the molecular changes during breast cancer immunotherapy and discover new biomarkers and therapeutic targets.
8.Ensure that all charts are clearly and accurately labeled, including complete statistical information and legend descriptions.
9.Add the latest relevant research literature to make the research more timely and persuasive.
10.Some paragraphs are rather redundant. Simplify the language to improve the readability of the article

***PART-2 Question and answer***

***For reviewer 1***

**Q1: Further elaborate on the current status of the cGAS-STING pathway in the field of breast cancer immunotherapy research, emphasizing the gaps filled by this study**

A1: Thanks for your comments. We have added more details about cGAS-STING pathway in BRCA immunotherapy research, and clarified the new progressions in our research. Here we show the part of our revisions and please check the details in revised manuscript:

*“……It suppresses tumors by activating type I interferons (IFNs), triggered by cytosolic DNA from chromosomal instability (CIN) or therapy-induced damage. This enhances dendritic cell infiltration and CD8⁺ T cell activation, boosting antitumor immunity. Tumor-derived DNA or cGAMP also stimulates IFN release in immune cells like NK cells [1-7]. Conversely, in high-CIN tumors, such as triple-negative breast cancer, persistent cGAS-STING signaling promotes progression via NF-κB-mediated IL-6 production, STAT3 activation, and PD-L1 upregulation, aiding immune evasion[8-10]. Further research is needed to clarify this pathway’s complex dynamics……”*

**Q2: Provide detailed parameter settings of each machine-learning algorithm to enhance the reproducibility of the methods.**

A2: Thanks for your comments. We have described more about the machine-learning, and we have uploaded our R codes in our first submission. Here, we show the revised description and R codes:

*“Machine learning: Support Vector Machine(SVM), RandomForest(RF), eXtreme Gradient Boosting(XGB), Generalized Linear Model(GLM), Gradient Boosting Machine(gbm), K-Nearest Neighbors(KKNN), Neural Network(NNET), Least Absolute Shrinkage and Selection Operator(LASSO) were applied in this study, and each algorithm used 10-fold repeated cross-validation. Here, algoritms from R including rf, svmRadial, xgbDART, glm, gbm, kknn, glmnet were applied, and all of them were performed by “caret” package. In machine learning part, TCGA cohort was applied as training cohort, and 70% of which was intra-training subgroup, and other 30% was intra-testing subgroup. Details about the R codes were in supplementary files.”*

**R code:**

*library(caret)*

*library(DALEX)*

*library(ggplot2)*

*library(randomForest)*

*library(kernlab)*

*library(xgboost)*

*library(pROC)*

*set.seed(123)*

*inputFile="exp.txt"*

*geneFile="gene.txt"*

*setwd("/Users/Desktop/miR-sGAS-STING ")*

*data=read.table(inputFile, header=T, sep="\t", check.names=F, row.names=1)*

*geneRT=read.table(geneFile, header=F, sep="\t", check.names=F)*

*data=data[as.vector(geneRT[,1]),]*

*row.names(data)=gsub("-", "_", row.names(data))*

*data=t(data)*

*group=gsub("(.*)\\_(.*)", "\\2", row.names(data))*

*data=as.data.frame(data)*

*data$Type=group*

*inTrain<-createDataPartition(y=data$Type, p=0.7, list=F)*

*train<-data[inTrain,]*

*test<-data[-inTrain,]*

*library(openxlsx)*

*write.xlsx (train,file="train.xlsx",sep="\t",quote=F,colnames=T)*

*write.xlsx (test,file="test.xlsx",sep="\t",quote=F,colnames=T)*

*test <- read.xlsx("/Users/jeric/Desktop/miR-STING/F4/construction/test1.xlsx")*

*train <- read.xlsx("/Users/jeric/Desktop/miR-STING/F4/construction/train1.xlsx")*

*control=trainControl(method="repeatedcv", number=10, savePredictions=TRUE)*

*mod_rf = train(Type ~ ., data = train, method='rf', trControl = control)*

*mod_svm=train(Type ~., data = train, method = "svmRadial", prob.model=TRUE, trControl=control)*

*mod_xgb=train(Type ~., data = train, method = "xgbDART", trControl=control)*

*mod_glm=train(Type ~., data = train, method = "glm", family="binomial", trControl=control)*

*mod_gbm=train(Type ~., data = train, method = "gbm", trControl=control)*

*mod_kknn=train(Type ~., data = train, method = "kknn", trControl=control)*

*mod_nnet=train(Type ~., data = train, method = "nnet", trControl=control)*

*mod_lasso=train(Type ~., data = train, method = "glmnet", trControl=control)*

*p_fun=function(object, newdata){*

*predict(object, newdata=newdata, type="prob")[,2]}*

*yTest=ifelse(test$Type=="control", 0, 1)*

*explainer_rf=explain(mod_rf, label = "RF",data = test[,-ncol(test)], y = yTest, predict_function = p_fun, verbose = FALSE)*

*mp_rf=model_performance(explainer_rf)*

*explainer_svm=explain(mod_svm, label = "SVM",data = test[,-ncol(test)], y = yTest,predict_function = p_fun,verbose = FALSE)*

*mp_svm=model_performance(explainer_svm)*

*explainer_xgb=explain(mod_xgb, label = "XGB",data = test[,-ncol(test)], y = yTest,predict_function = p_fun,verbose = FALSE)*

*mp_xgb=model_performance(explainer_xgb)*

*explainer_glm=explain(mod_glm, label = "GLM",data = test[,-ncol(test)], y = yTest,predict_function = p_fun,verbose = FALSE)*

*mp_glm=model_performance(explainer_glm)*

*explainer_gbm=explain(mod_gbm, label = "GBM",data = test[,-ncol(test)], y = yTest,predict_function = p_fun,verbose = FALSE)*

*mp_gbm=model_performance(explainer_gbm)*

*explainer_kknn=explain(mod_kknn, label = "kknn",data = test[,-ncol(test)], y = yTest,predict_function = p_fun,verbose = FALSE)*

*mp_kknn=model_performance(explainer_kknn)*

*explainer_nnet=explain(mod_nnet, label = "nnet",data = test[,-ncol(test)], y = yTest,predict_function = p_fun,verbose = FALSE)*

*mp_nnet=model_performance(explainer_nnet)*

*explainer_lasso=explain(mod_lasso, label = "lasso",data = test[,-ncol(test)], y = yTest,predict_function = p_fun,verbose = FALSE)*

*mp_lasso=model_performance(explainer_lasso)*

*dev.off()*

*pred1=predict(mod_rf, newdata=test, type="prob")*

*pred2=predict(mod_svm, newdata=test, type="prob")*

*pred3=predict(mod_xgb, newdata=test, type="prob")*

*pred4=predict(mod_glm, newdata=test, type="prob")*

*pred5=predict(mod_gbm, newdata=test, type="prob")*

*pred6=predict(mod_kknn, newdata=test, type="prob")*

*pred7=predict(mod_nnet, newdata=test, type="prob")*

*pred8=predict(mod_lasso, newdata=test, type="prob")*

*roc1=roc(yTest, as.numeric(pred1[,2]))*

*roc2=roc(yTest, as.numeric(pred2[,2]))*

*roc3=roc(yTest, as.numeric(pred3[,2]))*

*roc4=roc(yTest, as.numeric(pred4[,2]))*

*roc5=roc(yTest, as.numeric(pred5[,2]))*

*roc6=roc(yTest, as.numeric(pred6[,2]))*

*roc7=roc(yTest, as.numeric(pred7[,2]))*

*roc8=roc(yTest, as.numeric(pred8[,2]))*

*pdf(file="ROC2.pdf", width=5, height=5)*

*plot(roc1, print.auc=F, legacy.axes=T, main="", col="#dc5165")*

*plot(roc2, print.auc=F, legacy.axes=T, main="", col="#56acda", add=T)*

*plot(roc3, print.auc=F, legacy.axes=T, main="", col="#efd466", add=T)*

*plot(roc4, print.auc=F, legacy.axes=T, main="", col="#7bc6a9", add=T)*

*plot(roc5, print.auc=F, legacy.axes=T, main="", col="#e482c0", add=T)*

*plot(roc6, print.auc=F, legacy.axes=T, main="", col="#F4A582", add=T)*

*plot(roc7, print.auc=F, legacy.axes=T, main="", col="pink", add=T)*

*plot(roc8, print.auc=F, legacy.axes=T, main="", col="grey", add=T)*

*legend('bottomright',*

*c(paste0('RF: ',sprintf("%.03f",roc1$auc)),*

*paste0('SVM: ',sprintf("%.03f",roc2$auc)),*

*paste0('XGB: ',sprintf("%.03f",roc3$auc)),*

*paste0('GLM: ',sprintf("%.03f",roc4$auc)),*

*paste0('GBM: ',sprintf("%.03f",roc5$auc)),*

*paste0('KKNN: ',sprintf("%.03f",roc6$auc)),*

*paste0('NNET: ',sprintf("%.03f",roc7$auc)),*

*paste0('LASSO: ',sprintf("%.03f",roc8$auc))),*

*col=c("#dc5165", "#56acda", "#efd466","#7bc6a9","#e482c0", "#F4A582","pink","grey"), lwd=3, bty = 'n')*

*dev.off()*

*all_data<-read.xlsx("/Users/Desktop/miR-STING/F3/AI/identification/select.xlsx")*

*train_data <- all_data %>% slice_sample(prop = .75)*

*write.xlsx (train_data,file="train.xlsx",sep="\t",quote=F,colnames=T)*

*train_data <- read.xlsx("/Users/Desktop/miR-STING/F3/AI/identification/train.xlsx")*

*test_data <- anti_join(all_data,train_data,by="IKZF1")*

*write.xlsx (test_data,file="test.xlsx",sep="\t",quote=F,colnames=T)*

*test_data <- read.xlsx("/Users/jeric/Desktop/miR-STING/F3/AI/identification/test.xlsx")*

*test_data <- read.xlsx("/Users/jeric/Desktop/miR-STING/F3/AI/identification/train.xlsx")*

*train_data <- read.xlsx("/Users/jeric/Desktop/miR-STING/F3/AI/identification/test.xlsx")*

*pdf(file="ROC1.pdf", width=3.5, height=3.5)*

*g1<-ggroc(roc1, legacy.axes = TRUE, color = "#dc5165", size = 1)*

*g1 + theme_bw() +*

*geom_segment(aes(x = 0, xend = 1, y = 0, yend = 1), color="Grey",*

*linetype=1)+*

*scale_x_continuous(expand = c(0.02,0.02))+*

*scale_y_continuous(expand = c(0.02,0.02))+*

*xlab("1-Specificity")+*

*ylab("Sensitivity")*

*dev.off()*

*inTrain<-createDataPartition(y=data$Type, p=0.7, list=F)*

*train<-data[inTrain,]*

*test<-data[-inTrain,]*

*write.xlsx (train,file="train.xlsx",sep="\t",quote=F,colnames=T)*

*write.xlsx (test,file="test.xlsx",sep="\t",quote=F,colnames=T)*

*control=trainControl(method="repeatedcv", number=5, savePredictions=TRUE)*

*mod_rf = train(Type ~ ., data = train, method='rf', trControl = control)*

*mod_glm=train(Type ~., data = train, method = "glm", family="binomial", trControl=control)*

*mod_gbm=train(Type ~., data = train, method = "gbm", trControl=control)*

*p_fun=function(object, newdata){*

*predict(object, newdata=newdata, type="prob")[,2]}*

*yTest=ifelse(test$Type=="LR", 0, 1)*

*explainer_rf=explain(mod_rf, label = "RF",data = test[,-ncol(test)], y = yTest,predict_function = p_fun,verbose = FALSE)*

*mp_rf=model_performance(explainer_rf)*

*explainer_glm=explain(mod_glm, label = "GLM",data = test[,-ncol(test)], y = yTest,predict_function = p_fun,verbose = FALSE)*

*mp_glm=model_performance(explainer_glm)*

*explainer_gbm=explain(mod_gbm, label = "GBM",data = test[,-ncol(test)], y = yTest,predict_function = p_fun,verbose = FALSE)*

*mp_gbm=model_performance(explainer_gbm)*

*pred1=predict(mod_rf, newdata=test, type="prob")*

*pred4=predict(mod_glm, newdata=test, type="prob")*

*pred5=predict(mod_gbm, newdata=test, type="prob")*

*roc1=roc(yTest, as.numeric(pred1[,2]))*

*roc4=roc(yTest, as.numeric(pred4[,2]))*

*roc5=roc(yTest, as.numeric(pred5[,2]))*

*pdf(file="ROC.pdf", width=5, height=5)*

*plot(roc1, print.auc=F, legacy.axes=T, main="", col="#dc5165")*

*plot(roc4, print.auc=F, legacy.axes=T, main="", col="#7bc6a9", add=T)*

*plot(roc5, print.auc=F, legacy.axes=T, main="", col="#e482c0", add=T)*

*legend('bottomright',*

*c(paste0('RF: ',sprintf("%.03f",roc1$auc)),*

*paste0('GLM: ',sprintf("%.03f",roc4$auc)),*

*paste0('GBM: ',sprintf("%.03f",roc5$auc))),*

*col=c("#dc5165", "#7bc6a9","#e482c0"), lwd=5, bty = 'n')*

*dev.off()*

*library(openxlsx)*

*library(randomForest)*

*library(caret)*

*library(DALEX)*

*library(ggplot2)*

*library(randomForest)*

*library(kernlab)*

*library(xgboost)*

*library(pROC)*

*setwd("/Users/Desktop/miR-STING")*

*train <- read.xlsx("/Users/ Desktop/miR-STING/F4/clinical_data/gide_cohort/gide_1/TCGA-BRCA.xlsx")*

*control=trainControl(method="repeatedcv", number=5, savePredictions=TRUE)*

*mod_rf = train(Type ~ ., data = train, method='rf', trControl = control)*

*test <- read.xlsx("/Users/jeric/Desktop/miR-STING/F4/clinical_data/gide_cohort/gide_2/gide_2_QPC.xlsx")*

*pred1=predict(mod_rf, newdata=test, type="raw")*

*write.xlsx (pred1,file="GROUP-GIDE_2.xlsx",sep="\t",quote=F,colnames=T)*

**Q3: In the cell experiment section, supplement details such as the number of cell culture passages and the detection of transfection efficiency.**

A3: Thanks for your comments. We have described our methods about experiments section more carefully. Here we show part of the revised manuscript:

***“Transwell Migration and EdU Proliferation Assays:*** *For migration assays, BRCA cell lines were treated with different, followed by PBS washing, and then digested with EDTA-containing trypsin for 2 minutes, neutralizing the trypsin with complete medium. Centrifuge at 100g for 5 minutes, collect the cell pellet, resuspend the cells in medium containing 2% FBS, and adjust the cell concentration to* $4\times{10}^{5}$*cells/ml. Trans-well cells were pre-treated with PBS for 2 hours. After that, 100ul* $4\times{10}^{5}$*cells/ml cells were seeded in the upper chamber of transwell inserts with an 8-µm pore size. The lower chamber contained a medium supplemented with 10% FBS as a chemoattractant. After 24 and 48 hours, non-migrated cells on the upper surface were removed and migrated cells on the lower surface were fixed with 4% paraformaldehyde, stained with 0.1% crystal violet, and counted under a microscope. For proliferation assays, cells with different treatments were washed with PBS 5 mins for twice, followed by being incubated with 10 µM EdU for another 2 hours, fixed with 4% paraformaldehyde, and stained using a Click-iT EdU imaging kit. Fluorescence signals were detected with a confocal microscope, and images were analyzed using ImageJ.”*

**Q4: When presenting the relationship between gene expression and immunotherapy response, increase the analysis of more clinical feature data.**

A4: Thanks for your comments. We have described our methods about experiments section more carefully. Here we show part of the revised manuscript:

*“Research also explored the roles of clinical parameters in HOXC13-regulated prognosis and anti-PD1/PDL1 response, and the results presents a comprehensive analysis of HOXC13 expression in relation to anti-PD-1 therapy response, TIDE scores, and overall survival (OS) across various tumor stages. In the Figure S3A, bar charts reveal that low HOXC13 expression is associated with better anti-PD-1 response, particularly in T1 (p=0.0233, ~70% low in responders), N3 (p=0.0416, ~80% low), and Age<=50 (p=0.0386, ~70% low). The Figure S3B, using scatter plots, shows that low HOXC13 expression correlates with lower TIDE scores (indicating better immunotherapy response) in T1 (p=0.0123), N3 (p=0.0102), Stage I (p=0.0047). The Figure S3C, with Kaplan-Meier survival curves, demonstrates that low HOXC13 expression is linked to improved OS in T2 (p=1.536E-01, ~0.6 vs. ~0.4 at 25 years), T3 (p=1.708E-02, ~0.5 vs. ~0.3), N1 (p=3.931E-02, ~0.5 vs. ~0.3), N2 (p=3.452E-02, ~0.5 vs. ~0.3), M0 (p=2.294E-01, ~0.5 vs. ~0.3). Collectively, these findings suggest that low HOXC13 expression may serve as a predictive biomarker for enhanced anti-PD-1 therapy response and better survival outcomes, particularly in early-stage cancer patients.”*

**
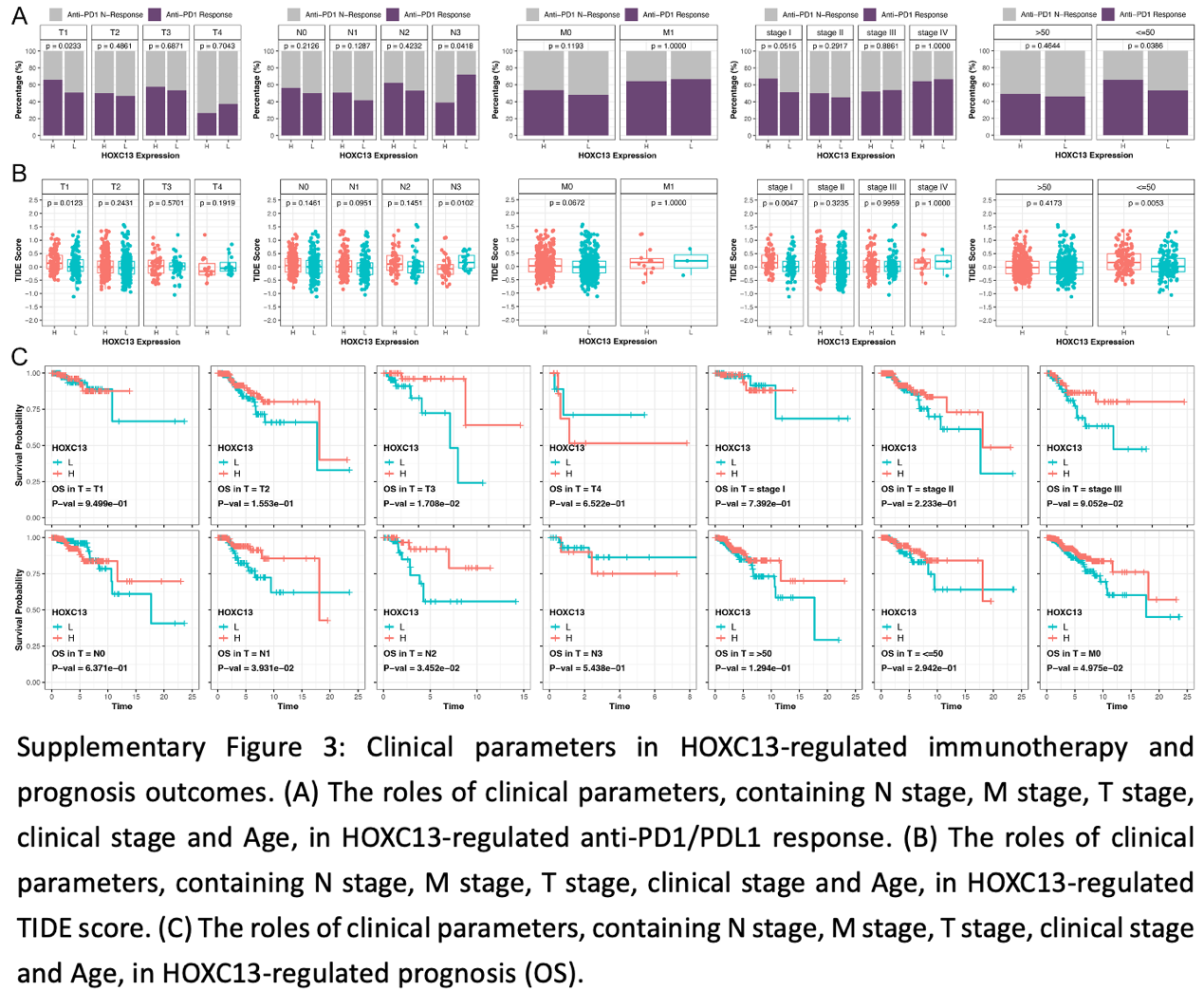
**

**Q5: In the weighted gene co - expression network analysis (WGCNA), in addition to identifying gene modules related to immune response and subgroup classification, further explore the interaction mechanisms of genes within the module. For example, construct a gene regulatory network to clarify the upstream and downstream relationships between core genes and other genes, and excavate potential regulatory pathways, providing a more comprehensive perspective for understanding the molecular mechanism of breast cancer immunotherapy.**

A5: Thanks for your comments. We have performed gene interaction map and signaling pathway analysis as supplementary data (Figure S4) in manuscript. Here we showed the data:

*“We explore the roles of genes in module lightcyan by enriched analysis and correlation analysis. The results showed a comprehensive analysis of protein-protein interaction (PPI) networks and their functional enrichment for genes associated with immune responses. Figure S4A illustrates a dense PPI network with key hub genes such as PTPN7, CD3E, CD247, CD5, SLA2, SIRPG, LCK, and ITK, highlighted in orange, indicating their central roles in the network. These genes are interconnected with numerous other proteins, suggesting their involvement in critical immune-related pathways. Figure S4B zooms into a hub-gene map, focusing on specific interactions among genes like UBASH3A, PTPN7, CD3E, CD247, SLA2, SIRPG, LCK, ITK, SLAMF1, SLAMF6, CD5, LY9, and TRAV3, with connections to T-cell receptor (TCR) signaling components (TRBV5-4, TRAV12-3, TRDV1), emphasizing their roles in T-cell mediated immunity. Figure S4C displays KEGG pathway enrichment, identifying significant pathways such as Th17 cell differentiation, ECM-receptor interaction, Leishmaniasis, and Type I diabetes mellitus, with gene counts ranging from 60 to 120 and P-values as low as 4.7e-40, indicating strong statistical significance. The rich factor, ranging from 0.4 to 0.8, highlights the proportion of genes involved in these pathways. Figure S4D shows Reactome enrichment, revealing pathways like extracellular matrix organization, signaling by interleukins, and GPCR ligand binding, with gene counts between 60 and 150 and P-values from 1.2e-54 to 9.6e-16, underscoring their relevance to immune and structural functions. Finally, Figure S4E depicts Gene Ontology (GO) enrichment, focusing on biological processes such as cell chemotaxis, extracellular structure organization, and immune response regulation, with gene counts from 180 to 220 and P-values ranging from 7.8e-68 to 1.4e-47, reflecting the diverse roles of these genes in immune modulation, leukocyte migration, and cellular adhesion. Collectively, these results suggest that the identified hub genes and their associated pathways play pivotal roles in immune regulation, T-cell signaling, and inflammatory responses, providing potential targets for therapeutic interventions in immune-related diseases.”*

**
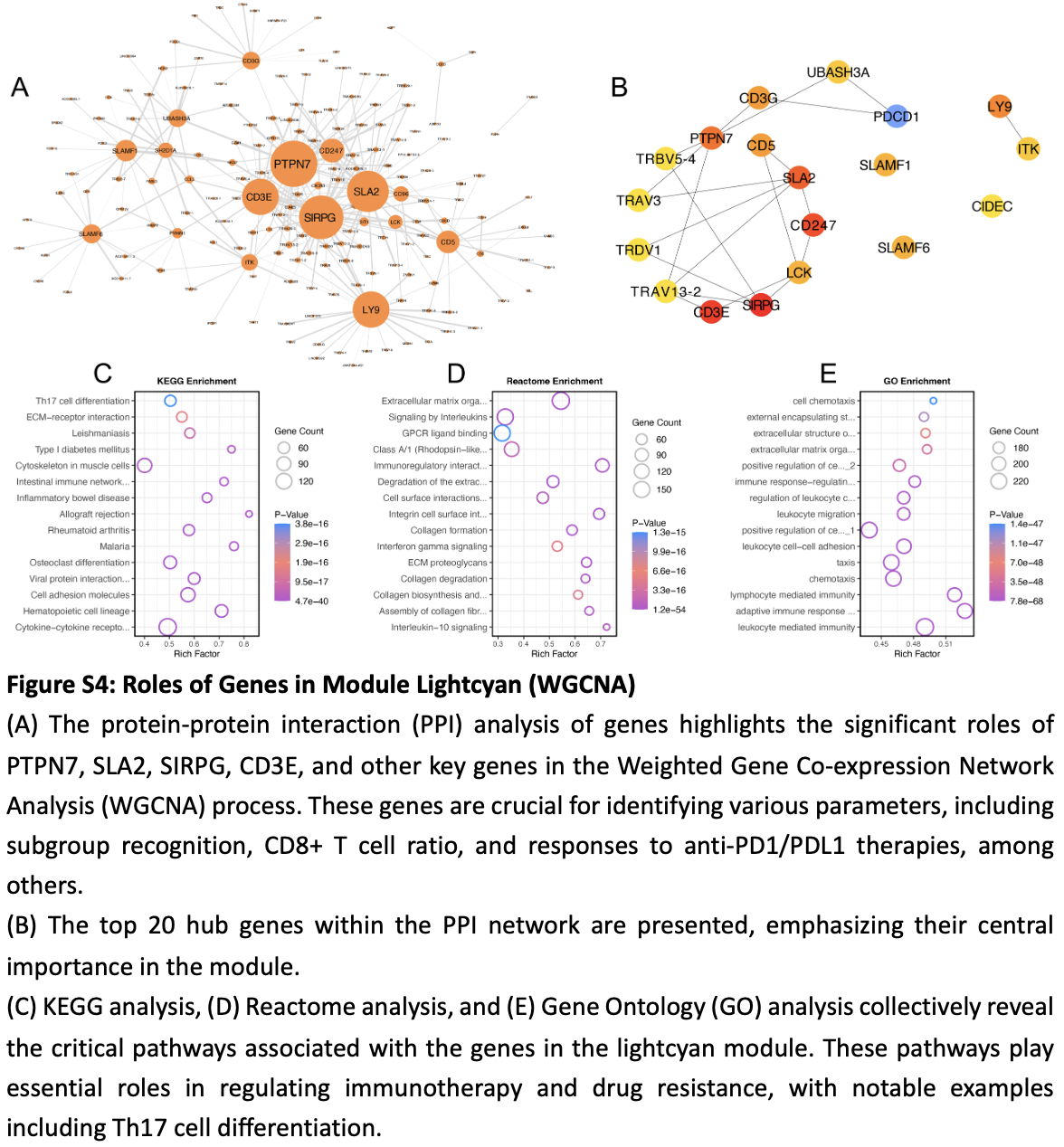
**

**Q6: Currently, the research mainly relies on in vitro experiments to verify the function of HOXC13. In-vivo animal experiments should be carried out. Establish a breast cancer mouse model, and by knocking down or overexpressing the HOXC13 gene, observe tumor growth, immune cell infiltration, and the response to immunotherapy drugs to verify its role in the in - vivo environment and enhance the clinical translation value of the research results.**

A6: Thanks for your comments, we have performed the experiments in vivo since your manuscript submission, for further research about mechanisms of HOXC13 regulating BRCA immune escape, which were included in another independent project. However, we displayed our results here to verify our conclusions in this manuscript:

*In our following research, we explore the down-regulator of HOXC13, and we have constructed HOXC13 over-expression cell line (4T1), and establish a breast cancer mouse model. The results showed that down-regulation of HOXC13 restored the T cell function (7.12% vs 12.5%).*

**
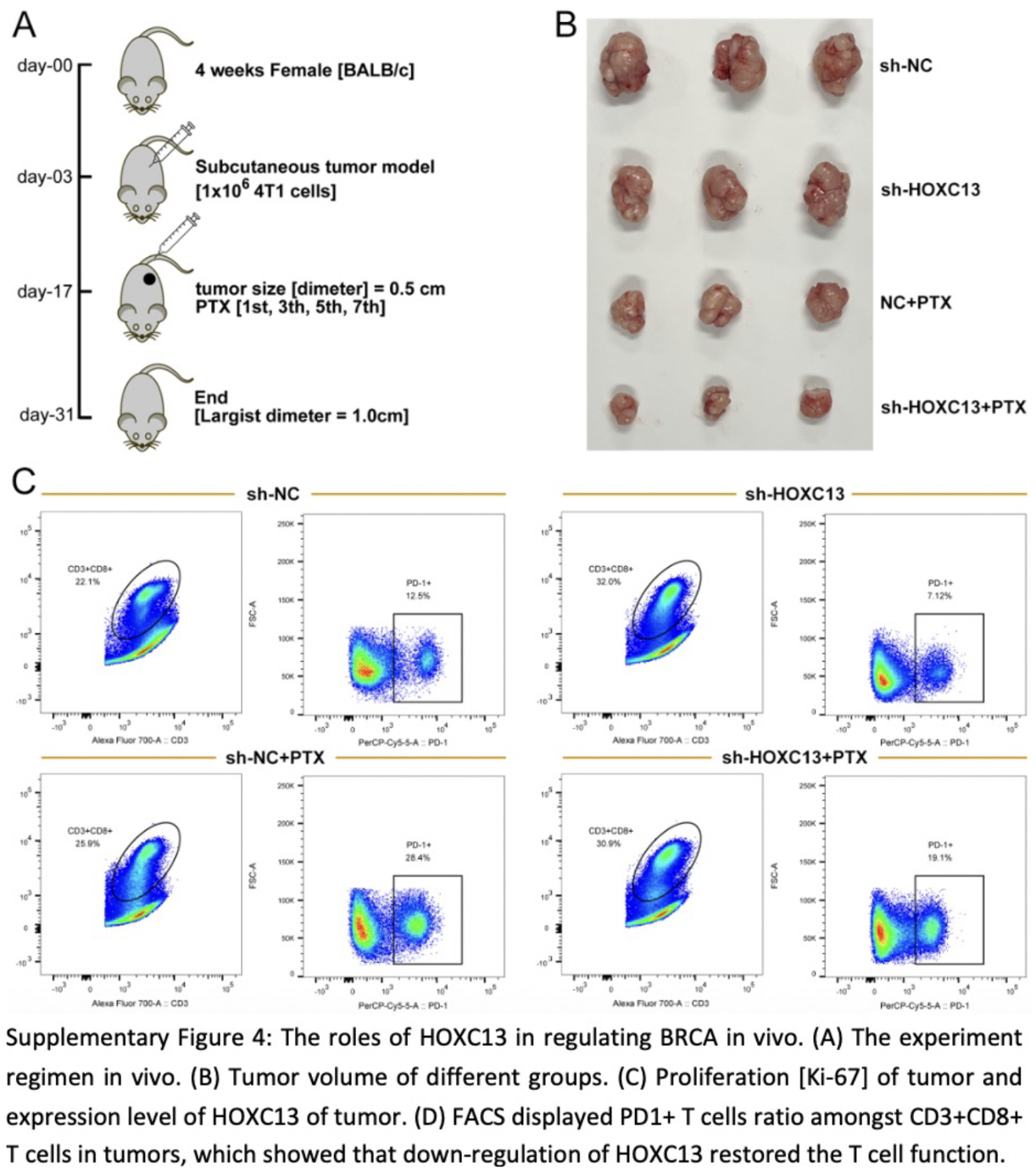
**

**Q7: This research is mainly based on transcriptome data. Proteomics, metabolomics, and other multi - omics data can be integrated. Proteomics data can directly reflect the final products of gene expression, and metabolomics data can reveal cell metabolic changes. Multi-omics joint analysis helps to more deeply understand the molecular changes during breast cancer immunotherapy and discover new biomarkers and therapeutic targets.**

A7: Thanks for your comments. In this study we focus on genes expression in disease treatment, such as multi-gene-based prognosis prediction model, anti-PD1 response prediction model and drug sensitivity prediction model. Our research is designed to construct more clinical treatment related Decision-Making Tools, which are like with 21-gene detection (a decision-making tools for early breast cancer to make chemotherapy regimen). Inaddition, we want to further clarified the potential mechanisms of HOXC13 about regulating immunotherapy resistance and drug resistance in BRCA by experiments in vitro. However, we understand your concerns, we explore the protein expression of Hoxc13 in BRCA and adjacent tissues, and also explore the correlation between protein level and clinical parameters (*This part of work not put into revised manuscript*):


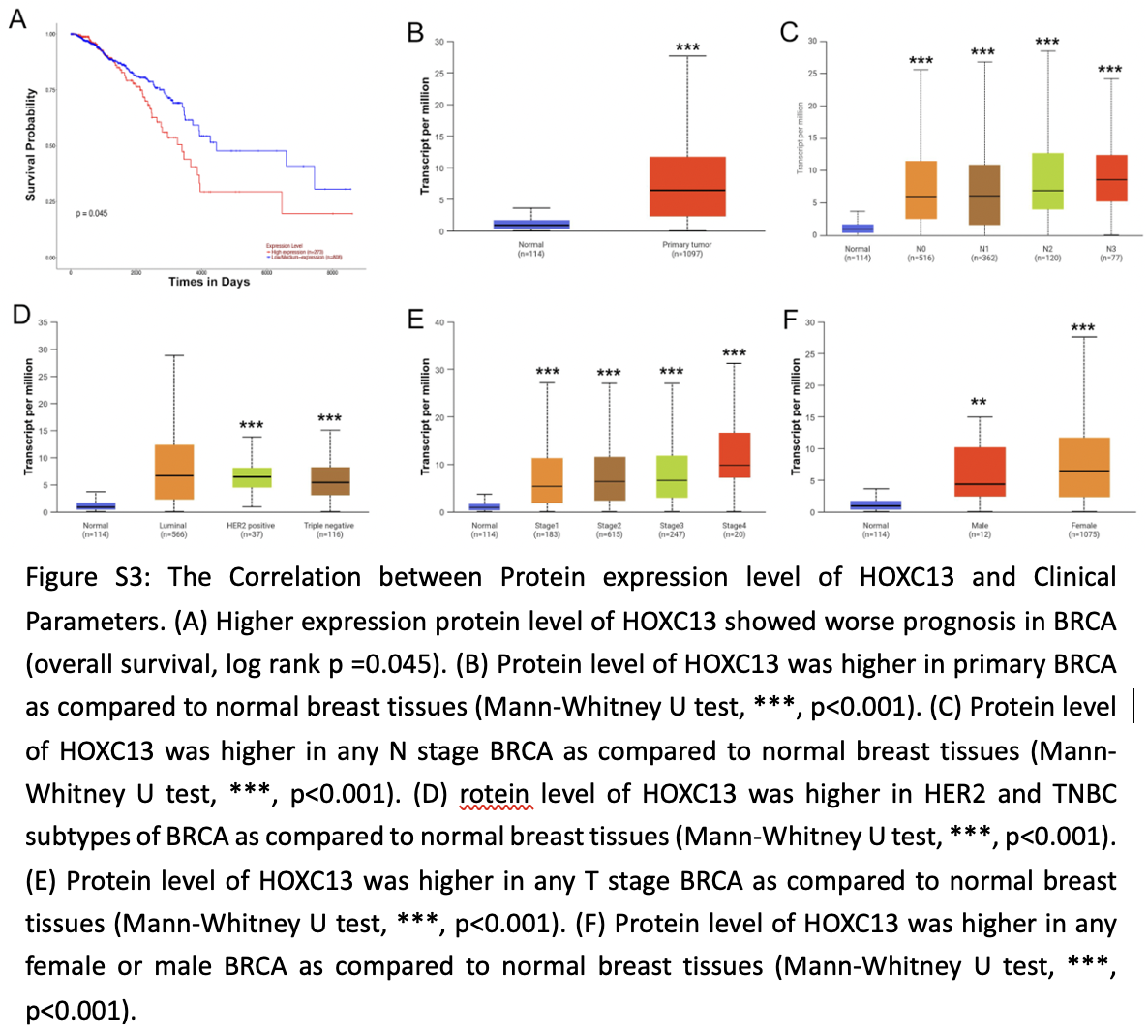


**Q8: Ensure that all charts are clearly and accurately labeled, including complete statistical information and legend descriptions.**

A8: Thanks for your comments, all figures and tables were clearly and accurately labeled, and we check the figure legends carefully again. If there is anything unreasonable in our description, please point it out directly, and we will make point-to-point revisions.

**Q9: Add the latest relevant research literature to make the research timelier and persuasive.**

A9: Thanks for your comments, we have added latest relevant literature, please check the manuscript.

**Q10: Some paragraphs are rather redundant. Simplify the language to improve the readability of the article**

A10: Thanks for your comments, we have simplified our paragraphs, please check them in manuscript. Here we show part of the revised manuscript:

*“……Our study combines bioinformatics and experimental validation to pinpoint novel biomarkers and therapeutic targets for breast cancer, focusing on high-risk subtypes like TNBC. We identified subtype-specific immune landscapes and the miR-26a-5p/HOXC13 axis as critical immune response regulators. Patients with elevated HOXC13 or immune-excluded subtypes may respond poorly to checkpoint inhibitors alone, suggesting combination therapies targeting the JAK-STAT pathway, HOXC13, or miR-26a-5p restoration. Additionally, machine learning models enhance our ability to predict immunotherapy responses, advancing precision oncology by enabling better patient stratification and treatment planning.*

*However, limitations temper our findings. The retrospective analysis requires prospective clinical studies to validate these biomarkers and subtypes. While the miR-26a-5p/HOXC13 axis showed promise in vitro, in vivo studies are essential to confirm its therapeutic potential and immune interactions. Relying on TIDE scores for immune dysfunction assessment calls for broader profiling with markers like tumor mutational burden and spatial immune data. The cGAS-STING pathway’s dual role in immunity also needs further exploration. Despite these challenges, our work lays a groundwork for personalized immunotherapies, aiming to boost treatment success for breast cancer patients……”*

**references**

[1] B. Zhang, M. Li, J. Ji, X. Si, X. Yin, G. Ji, L. Ren, H. Yao, A syringeable immunotherapeutic hydrogel enhances T cell immunity via in-situ activation of STING pathway for advanced breast cancer postoperative therapy, Front Immunol, 16 (2025) 1523436.

[2] Z. Gohari, L. Stojanovic, F.V. Rassool, Combining STING Agonists with PARP Inhibitors Mounts an NK-Dependent Defense against Therapy-Resistant Breast Cancer, Cancer Res, 85 (2025) 1747-1749.

[3] B. Ding, X. Liu, Z. Li, X. Xie, J. Li, J. Wang, S. Li, P. Wang, Y. Xie, X. Ma, H. Wang, C. Xie, X. Qiao, Y. Wang, J. Xu, Y. Feng, J. Hao, A novel platinum(IV) prodrug, gramine-Pt(IV) enhances chemoimmunotherapy by activating cGAS-STING and modulating TGF-β-MHC-I axis, Drug Resist Updat, 81 (2025) 101252.

[4] S. Xian, X. Chen, S. Ren, X. Chen, H. Wang, Ionizable STING-Activating Nanoadjuvants Enhance Tumor Immunogenicity and Potentiate Immunotherapy Efficacy in Solid Tumors, Cancer Res, 84 (2024) 3044-3057.

[5] T.L. Sheehy, A.J. Kwiatkowski, K. Arora, B.R. Kimmel, J.A. Schulman, K.N. Gibson-Corley, J.T. Wilson, STING-Activating Polymer-Drug Conjugates for Cancer Immunotherapy, ACS Cent Sci, 10 (2024) 1765-1781.

[6] X. Cheng, C. Yu, Y. Zhang, Y. Peng, Y. Liu, H. Fa, L. Xia, L. Qin, S. Guan, X. Wu, J. Wu, Y. Wang, J. Liu, L. Sun, J. Liang, Y. Shang, Loss of ZNF408 attenuates STING-mediated immune surveillance in breast carcinogenesis, iScience, 27 (2024) 110276.

[7] J.H. Maltbaek, S. Cambier, J.M. Snyder, D.B. Stetson, ABCC1 transporter exports the immunostimulatory cyclic dinucleotide cGAMP, Immunity, 55 (2022) 1799-1812.e1794.

[8] S.F. Bakhoum, B. Ngo, A.M. Laughney, J.A. Cavallo, C.J. Murphy, P. Ly, P. Shah, R.K. Sriram, T.B.K. Watkins, N.K. Taunk, M. Duran, C. Pauli, C. Shaw, K. Chadalavada, V.K. Rajasekhar, G. Genovese, S. Venkatesan, N.J. Birkbak, N. McGranahan, M. Lundquist, Q. LaPlant, J.H. Healey, O. Elemento, C.H. Chung, N.Y. Lee, M. Imielenski, G. Nanjangud, D. Pe'er, D.W. Cleveland, S.N. Powell, J. Lammerding, C. Swanton, L.C. Cantley, Chromosomal instability drives metastasis through a cytosolic DNA response, Nature, 553 (2018) 467-472.

[9] C. Hong, M. Schubert, A.E. Tijhuis, M. Requesens, M. Roorda, A. van den Brink, L.A. Ruiz, P.L. Bakker, T. van der Sluis, W. Pieters, M. Chen, R. Wardenaar, B. van der Vegt, D.C.J. Spierings, M. de Bruyn, M. van Vugt, F. Foijer, cGAS-STING drives the IL-6-dependent survival of chromosomally instable cancers, Nature, 607 (2022) 366-373.

[10] H. Vasiyani, M. Mane, K. Rana, A. Shinde, M. Roy, J. Singh, D. Gohel, F. Currim, R. Srivastava, R. Singh, DNA damage induces STING mediated IL-6-STAT3 survival pathway in triple-negative breast cancer cells and decreased survival of breast cancer patients, Apoptosis, 27 (2022) 961-978.

**From corresponding author: Wang Jingshuai**

**Institute: Department of obstetrics and gynecology, shanghai east hospital, Tongji university school of medicine**

**E-mail: wangjs90@126.com**
